# DiMMPose: A Diffusion-Mamba Hybrid Framework with Multi-Prompt for Efficient and Robust 3D Human Pose Estimation

**DOI:** 10.3390/s26175559

**Published:** 2026-09-01

**Authors:** Xu Li, Xuefeng Guan, Chang Liu, Zengjie Wang, Xiaoyu Chen, Qingyang Xu, Shuyang Hou, Xiaopu Zhang, Huayi Wu

**Affiliations:** 1 State Key Laboratory of Information Engineering in Surveying, Mapping and Remote Sensing, Wuhan University, Wuhan 430079, China; oceanli@whu.edu.cn (X.L.); chang.l@whu.edu.cn (C.L.); zengjiewang@whu.edu.cn (Z.W.); 2018302140001@whu.edu.cn (X.C.); xuqingyang@whu.edu.cn (Q.X.); whuhsy@whu.edu.cn (S.H.); zhangxiaopu@whu.edu.cn (X.Z.); wuhuayi@whu.edu.cn (H.W.); 2 Hubei Luojia Laboratory, Wuhan 430079, China; 3 Collaborative Innovation Center of Geospatial Technology, Wuhan University, Wuhan 430079, China

**Keywords:** 3D human pose estimation (3DHPE), diffusion models, state-space models, mamba architecture, multi-prompt learning, spatiotemporal modeling

## Abstract

Monocular 3D Human Pose Estimation (3D HPE) typically adopts a two-stage approach: estimating 2D joint positions from images and then lifting them to 3D coordinates, effectively reducing dataset bias inherent in direct methods. However, current lifting techniques face two key challenges: many Transformer-based methods rely on attention-based or staged spatial–temporal modeling, which can limit efficient long-range frame-joint reasoning, while diffusion models support probabilistic modeling of pose uncertainty but remain sensitive to joint-coordinate noise. We propose DiMMPose, a diffusion-based framework enhanced by Mamba’s state-space model for robust and efficient 3D pose estimation. Its denoising process consists of two coordinated modules. The Spatiotemporal Mamba Block (STMB) serves as the core feature extraction module, employing internal Pose Mamba components with bidirectional state propagation and linear complexity to efficiently model long-range frame-joint dependencies. STMB further refines these features through Spatiotemporal Scan and Merge, which traverses the same skeleton tokens in complementary frame-joint orders and fuses the resulting representations. The Multi-Prompt Mamba Denoiser (MPMD) combines structured prompts encoded by LongCLIP with learnable prompt representations to provide anatomical and motion-related guidance during denoising. DiMMPose achieves an average MPJPE of 28.9 mm on Human3.6M under the DET setting, with action-specific errors of 21.2 mm for Walking and 22.0 mm for WalkTogether. It improves over FinePOSE by 3.0 mm, reduces inference latency by 57.1%, and achieves 23.0 mm MPJPE on MPI-INF-3DHP (N = 243).

## 1. Introduction

Monocular 3D Human Pose Estimation (3DHPE) seeks to accurately localize human joints in three-dimensional space from single-view images. It is a fundamental task underpinning numerous applications, including augmented and virtual reality, clinical diagnostics, rehabilitation, and human–robot interaction [1,2,3,4,5,6,7]. Current methodologies primarily encompass two categories: direct methods and two-stage methods. Direct methods map images directly to 3D poses in an end-to-end fashion [8,9], but inherently propagate image-domain noise and dataset biases into their predictions, resulting in limited generalization to diverse scenarios [10,11]. In contrast, two-stage methods first estimate intermediate 2D joint positions and then lift these predictions to 3D coordinates [3,12], achieving better robustness and stronger generalization capability [13,14]. In two-stage approaches, the lifting stage from 2D to 3D coordinates critically dictates the overall precision of the pose estimation [2,3].

**Figure 1:**
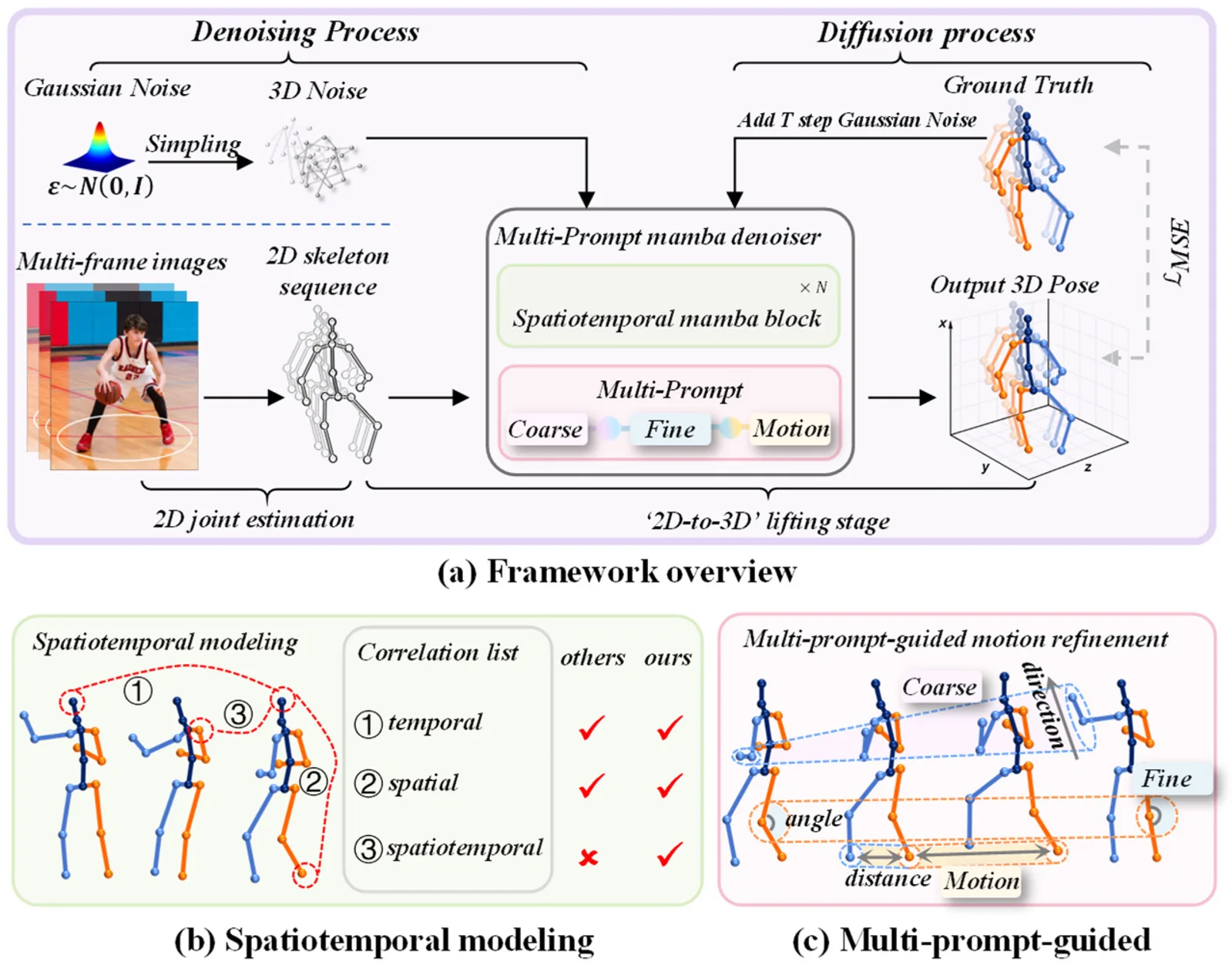
Overview of DiMMPose for 3D human pose estimation. (**a**) Framework overview: input 2D poses are lifted to 3D through a diffusion-based Multi-Prompt Mamba Denoiser that iteratively refines noisy poses over *T* steps, guided by spatiotemporal Mamba blocks and multi-level prompts (Coarse/Fine/Motion). (**b**) Spatiotemporal modeling: complementary frame-joint traversal and state-space propagation are used to capture temporal, spatial, and unified spatiotemporal correlations. (**c**) Multi-prompt guidance: three-level prompts (coarse for global motion, fine for joint details, and motion for dynamics) provide structured guidance during denoising for accurate 3D pose generation. Arrows indicate the processing direction; blue and orange skeleton segments distinguish the two body sides; ✓ and ✗ indicate whether the corresponding correlation is modeled, and the circled numbers refer to the correlation items shown in the diagram.

Existing lifting methods can be broadly categorized into two paradigms: deterministic regression and probabilistic generative modeling. Deterministic approaches, including recurrent models and Transformers, directly regress 3D coordinates from 2D inputs. recurrent neural networks (RNNs) and long short-term memory networks (LSTMs) [15,16] suffer from convergence instability and gradient vanishing when handling long temporal sequences. Regression-based Transformers [17,18,19] have recently gained traction due to their self-attention mechanisms, enabling simultaneous consideration of global spatial and temporal contexts and rapid convergence during training. However, their quadratic complexity O(*N*^2^) severely limits scalability to longer sequences [20,21,22], constraining real-time applications. In contrast, probabilistic generative approaches, particularly diffusion-based models [13,23,24], have gained prominence because iterative denoising can model pose uncertainty and generate multiple plausible hypotheses. As illustrated in Figure 1a, diffusion-based lifting transforms noisy 2D poses into precise 3D coordinates through *T*-step iterative refinement. Despite these advantages, the effectiveness of diffusion-based lifting fundamentally depends on two critical capabilities: robust spatiotemporal modeling to extract discriminative motion features that condition the denoising process, and stable iterative refinement mechanisms that remain robust to noise perturbations under ambiguous inputs.

These limitations manifest in two interconnected challenges that constrain diffusion-based lifting performance. First, existing spatiotemporal modeling approaches must balance effective frame-joint interaction with computational efficiency. While denoising Transformers model long-range dependencies through self-attention, their O(*N*^2^) complexity severely restricts scalability to extended sequences. Moreover, many architectures organize spatial and temporal modeling through separate or staged operations; although these designs can model both dimensions, efficiently integrating long-range frame-joint dependencies remains challenging. Second, diffusion-based denoising can remain sensitive to input noise and coordinate perturbations during iterative refinement. Under occluded or noisy 2D observations, additional semantic, anatomical, and motion-related cues may provide useful conditioning for resolving ambiguous skeletal configurations. Although multi-hypothesis sampling [25,26,27] partially alleviates uncertainty, it increases computational cost, motivating complementary structured guidance during denoising.

To address these challenges, we leverage state-space models (SSMs), particularly the Mamba architecture [28], which offers linear-complexity sequence modeling with a global receptive field. Bidirectional scanning strategies developed in visual Mamba variants further provide efficient long-sequence modeling for vision tasks [29,30]. Guided by these insights, we propose DiMMPose, a unified diffusion-based framework with two key innovations. To tackle the first challenge, we introduce the Spatiotemporal Mamba Block (STMB) as our core spatiotemporal modeling module. STMB is built upon the Pose Mamba Block (PMB), which captures long-range, cross-frame dependencies through bidirectional state-space scans with O(*N*) complexity. Building on this foundation, STMB enhances frame-joint modeling through Spatiotemporal Scan and Merge, which reorders the same skeleton tokens along complementary traversal paths, processes them through bidirectional state-space propagation, and fuses the resulting representations across scan directions. In this way, STMB model complementary temporal, spatial, and unified frame-joint dependencies without introducing separate learnable spatial and temporal branches. To tackle the second challenge, we design the Multi-Prompt Mamba Denoiser (MPMD), which introduces structured multi-level textual guidance into diffusion denoising. Inspired by vision-language models, MPMD integrates three hierarchical prompt categories encoded by LongCLIP [31]: coarse prompts capturing global motion semantics (e.g., walking speed, action type), fine-grained prompts encoding joint-level geometric details (e.g., elbow angle, knee flexion), and motion-status prompts representing dynamic state indicators (e.g., balance, body orientation). As shown in Figure 1c, these three-level prompts (Coarse/Fine/Motion) provide structured semantic, anatomical, and motion-related cues during denoising. Together with learnable prompt embeddings, they provide complementary conditioning for reverse diffusion, particularly when the 2D observations are noisy or ambiguous.

In summary, this paper makes the following contributions:

(i) We propose DiMMPose, a diffusion-Mamba framework that couples state-space spatiotemporal modeling with prompt-guided denoising for monocular 3D human pose estimation.

(ii) We introduce the Spatiotemporal Mamba Block (STMB), which models frame-joint dependencies through PMB and Spatiotemporal Scan and Merge for efficient skeleton-sequence representation.

(iii) We design the Multi-Prompt Mamba Denoiser (MPMD) to inject coarse, fine-grained, and motion-status prompts into the reverse diffusion process, providing structured conditioning for denoising under noisy or ambiguous 2D inputs.

(iv) We conduct comprehensive experiments on Human3.6M, MPI-INF-3DHP, synthetic occlusion, efficiency, and in-the-wild videos, showing consistent gains in accuracy, robustness, and computational efficiency.

Additionally, we will open the code after our research is published to further promote the development of related research. The code download website: https://github.com/ocean-xuli/DiMMPose (accessed on 27 August 2026).

## 2. Related Work

### 2.1. 3D Human Pose Estimation (3D HPE)

Three-dimensional Human Pose Estimation (3D HPE) aims to reconstruct 3D human joint positions from 2D images or video frames, serving as a fundamental component for applications ranging from action recognition to human–computer interaction [32,33,34]. Predominant approaches in this domain adopt a two-stage pipeline: first detecting 2D joint positions using established detectors [16,35], followed by lifting these 2D coordinates to 3D space through regression or optimization techniques [17,23,36,37,38]. This decomposition strategy effectively leverages abundant 2D annotations while mitigating the scarcity of 3D ground-truth data. This work specifically addresses the lifting stage, where the fundamental challenge lies in resolving depth ambiguity from 2D projections to accurate 3D reconstructions [17,36,39].

Recently, generative diffusion models [13,23,24,25] have emerged, which iteratively refine poses from noise or priors through progressive denoising, and thus enable multi-modal pose distribution modeling. However, existing diffusion-based methods can remain sensitive to coordinate noise during iterative refinement [23,25]. Multi-hypothesis sampling partially addresses uncertainty, but can substantially increase computational cost [25].

Early architectural approaches employed convolutional neural networks (CNNs) for extracting local joint features, effectively capturing neighboring joint relationships but struggling with long-range dependencies [4,40]. Subsequently, Transformers were introduced to capture global dependencies through self-attention mechanisms, achieving state-of-the-art performance [1,18,41]. While effective, Transformers suffer from quadratic complexity O(*N*^2^), severely limiting their scalability for long sequences [18,41]. Recent efforts have attempted to reduce this complexity through various strategies: PoseFormerV2 exploits frequency-domain representations to expand receptive fields efficiently [17], Hourglass Tokenizer [42] employs token pruning to reduce floating-point operations (FLOPs) by nearly 50%, and Uplift [43] utilizes temporally sparse inputs achieving 12× speedup. Recent studies also explore graph-based structures such as GLA-GCN [40] and GraphMLP [44], hybrid Transformer-GCN modeling such as MotionAGFormer [45], and kinematic or trajectory priors such as KTPFormer [18], providing complementary strategies for structural and motion modeling. Together, these developments motivate efficient mechanisms that can model long-range frame-joint dependencies while avoiding the quadratic cost of full self-attention.

### 2.2. State-Space Models in Vision

State-Space Models (SSMs) process sequential data through latent state transitions with linear computational complexity [46,47]. Unlike the pairwise token interactions used in self-attention, SSMs maintain compact hidden states that evolve along the sequence, providing an efficient basis for long-context modeling. This property has motivated the development of Mamba and its subsequent extensions for long-sequence representation learning.

Mamba introduces input-dependent selective state transitions and a hardware-aware parallel scan for efficient sequence modeling [28]. Subsequent surveys and vision applications further analyze and extend this state-space formulation [48,49]. Recent visual variants, including Vision Mamba (Vim) [29], VMamba [50], and VideoMamba [30], adapt selective scanning to image and video representations while retaining linear sequence modeling. In parallel, diffusion-based studies have explored temporal constraints and structured graph/set ordering [14,51], providing complementary mechanisms for incorporating structure during generative refinement.

Within 3D HPE, efficient temporal and structural modeling remains an active research topic [38,39]. Recent SSM-based methods include PoseMamba [52], STGJMamer [53], SAMA [54], and SasMamba [55], which explore bidirectional, graph-guided, structure-aware, or stride-based state-space modeling for pose estimation. These methods primarily follow deterministic regression-style pose prediction. Generative state-space modeling has also been explored in related articulated 3D generation; for example, MeshMamba [56] combines Mamba-based serialization with a diffusion model for articulated 3D mesh generation and reconstruction. This setting differs from the conditional monocular 2D-to-3D pose-lifting problem considered here. Accordingly, although state-space models have recently been introduced into 3D HPE and related 3D generation tasks, their integration into diffusion-based monocular 3D pose lifting has received limited attention. DiMMPose specifically integrates frame-joint state-space modeling into iterative diffusion denoising conditioned on 2D skeleton sequences, while retaining linear-complexity sequence modeling.

### 2.3. Prompt Learning for 3D HPE

Prompt learning guides vision models through semantic conditioning [57,58]. In 3D HPE, prompts embed joint-level or motion prior semantics into generative models, improving accuracy and smoothness of predictions [23,37,59]. Existing approaches utilize distinct prompt granularities. Fine-grained joint prompts encode anatomical constraints such as bone lengths and joint angles for precise localization [37]. Action-level prompts condition on motion categories to predict activity-specific patterns [59]. Temporal prompts maintain coherent sequences through transition patterns [23]. Each strategy demonstrates effectiveness in specific scenarios: fine-grained for static precision, action-level for activity recognition, and temporal for video tracking.

These studies show that prompt information can be introduced at different semantic levels, including local anatomical cues, action-level semantics, and temporal motion information. Recent work with LongCLIP [31] demonstrates enhanced capability for processing extended context. Motivated by the complementary roles of global, local, and temporal pose semantics, we organize the prompts in DiMMPose into three task-driven groups: coarse action/body cues, fine-grained anatomical cues, and motion-status cues. This grouping is not intended as an exhaustive or globally optimal decomposition; rather, it provides complementary conditioning whose individual contributions are evaluated in Section 4.5.4. Jointly optimized learnable prompt embeddings further provide adaptability beyond the predefined vocabulary.

## 3. Methodology

**Figure 2:**
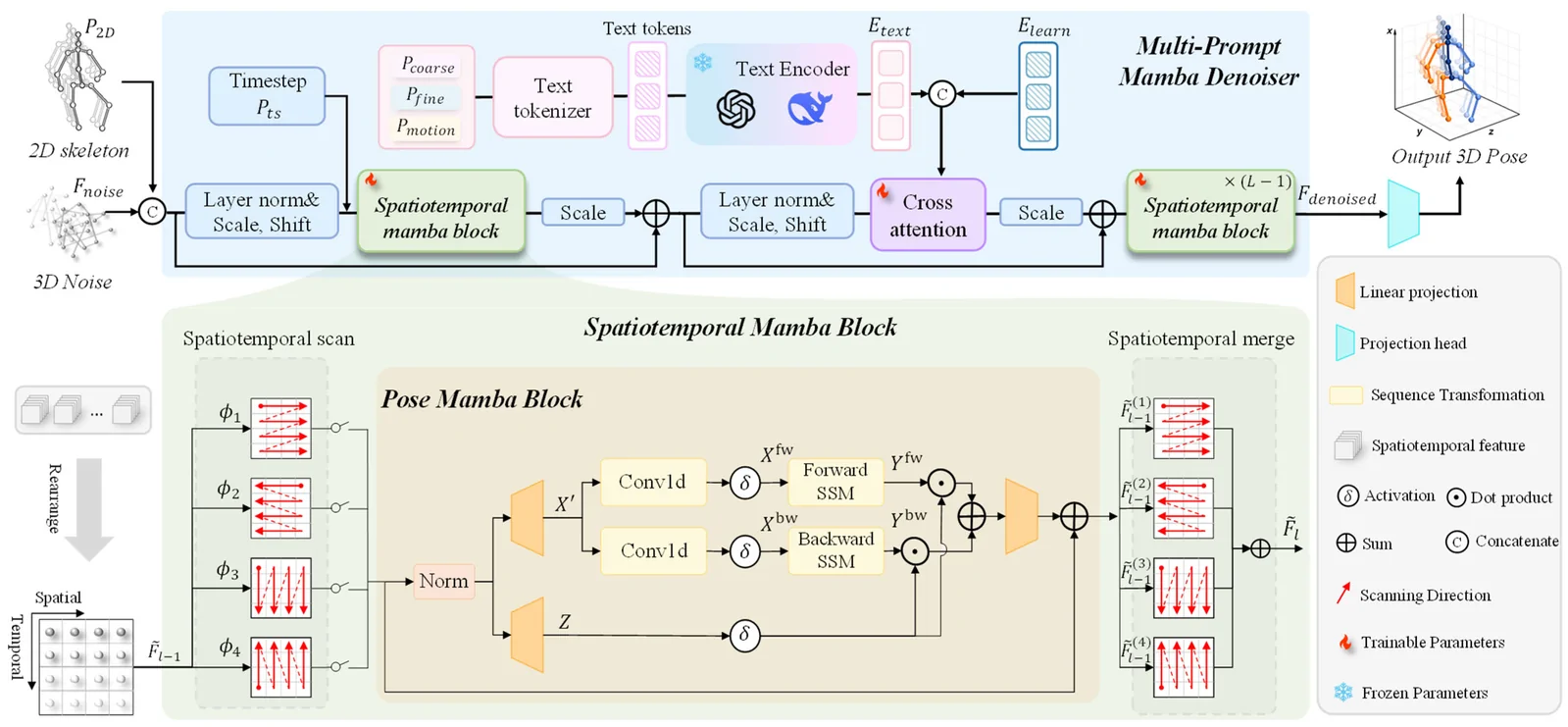
DiMMPose architecture overview. The Multi-Prompt Mamba Denoiser (MPMD, blue) performs conditional denoising on noisy skeletal features using the input 2D skeleton $P_{2D}$, multi-level prompts (coarse, fine-grained, and motion-status), and learnable prompt embeddings. The Spatiotemporal Mamba Block (STMB, green) captures frame-joint dynamics through Spatiotemporal Scan and Spatiotemporal Merge. Within STMB, the Pose Mamba Block (PMB, orange) uses bidirectional state-space modeling to capture long-range skeletal dependencies. The four labeled scan arrows in the STMB diagram indicate complementary frame-joint traversal orders; their PMB-processed features are subsequently fused through Spatiotemporal Merge.

As shown in Figure 2, DiMMPose is an efficient and robust framework for monocular 3D human pose estimation, which performs prompt-guided reverse diffusion from noisy 3D skeletal features $F_{\mathrm{noise}\;}$ under the condition of the input 2D skeleton sequence $P_{2D}$. Here, $P_{2D}$ is obtained from the frontend image-to-2D-skeleton pipeline. In the detected 2D setting, it is produced by a 2D pose detector, while in the ground-truth (GT) 2D setting, it corresponds to the dataset-provided 2D joint sequence. DiMMPose comprises two main modules: ① STMB, which employs state-space modeling with Spatiotemporal Scan and Spatiotemporal Merge mechanisms for unified frame-joint feature extraction with linear complexity, and ② MPMD, which integrates hierarchical prompts across three granularity levels, coarse, fine-grained, and motion-status, with learnable embeddings to guide the reverse denoising process.

### 3.1. Spatiotemporal Mamba Block (STMB)

As shown in the green part of Figure 2, the Spatiotemporal Mamba Block (STMB) is a critical component inside MPMD. It jointly propagates temporal dynamics across frames and spatial correlations among joints, enabling stable denoising of noisy skeletal features. STMB transforms the noisy skeletal feature $F_{\mathrm{noise}\;}\in R^{N\times J\times D}$, where N,J, and D denote the number of frames, joints, and feature dimension, respectively. The frame and joint dimensions are flattened as K=N×J, yielding an internal feature sequence $X\in R^{K\times D}$ for PMB processing. This flattening operation only serializes the frame-joint tokens for sequential state-space processing; it does not discard, pool, or aggregate any joint token. Here, X denotes the latent frame-joint feature sequence, while $P_{2D}$ denotes the observed 2D skeleton condition from the input pipeline.

#### 3.1.1. Pose Mamba Block

The Pose Mamba Block (PMB), a subcomponent of STMB as shown in the orange part of Figure 2, is a state-space unit designed to model the flattened frame-joint sequence with bidirectional propagation and linear computational complexity. It processes input features $X\in R^{K\times D}$ (where K=N×J is the flattened sequence length) to model long-range dependencies. The continuous-time dynamics are defined as:

(1) $$\frac{dh(t)}{dt}=Ah(t)+BX(t),Y(t)=Ch(t),$$

where $h(t)\in R^{D}$ is the latent state, $X(t)\in R^{D}$ is the input feature at time $t,Y(t)\in R^{D}$ is the output, and A,B,C are system matrices controlling state evolution, input influence, and output projection, respectively.

Discretization with Zero-Order Hold (ZOH) over the discretization interval Δt yields:

(2) $$h_{t}=\bar{A}h_{t-1}+\bar{B}X_{t},Y_{t}=Ch_{t},$$

where $\bar{A}=e^{A\Delta t}$ and $\bar{B}=(A\Delta t{)}^{-1}\left(e^{A\Delta t}-I\right)B$ are discretized matrices, ensuring efficient computation. Expanding Equation (2) recursively, the bidirectional outputs are derived to capture temporal context. The forward output at time step k integrates past information:

(3) $$Y_{k}^{fw}=\sum_{i=0}^{k}C_{fw}{\bar{A}}_{fw}^{k-i}{\bar{B}}_{fw}X_{i},$$

Similarly, the backward output incorporates future information by reversing the state propagation:

(4) $$Y_{k}^{bw}=\sum_{i=k}^{K-1}C_{bw}{\bar{A}}_{bw}^{i-k}{\bar{B}}_{bw}X_{i},$$

where $Y^{fw},Y^{bw}\in R^{K\times D_{1}}$ are the forward and backward feature maps, $D_{1}$ is the output dimension, and $C_{fw},C_{bw}$ are direction-specific output matrices. This bidirectional design synthesizes preceding and following skeletal context. During reverse denoising, each noisy frame-joint feature can therefore be refined using information propagated from both directions, supporting more temporally consistent skeletal representations across frames.

Feature fusion combines these outputs:

(5) $$X_{t}^{\prime },Z_{t}={Linear}^{X,Z}\left(Norm\left(F_{l-1}\right)\right),$$

(6) $$F_{l}^{\prime }=Linear\left(Y^{fw}\cdot f\left(Z_{t}\right)+Y^{bw}\cdot f\left(Z_{t}\right)\right),$$

(7) $$F_{l}=F_{l}^{\prime }+F_{l-1},$$

where f(⋅) is a ReLU activation, Norm(⋅) is layer normalization, and $F_{l}\in R^{K\times D}$ is the refined feature. This efficient long-range dependency modeling supports STMB’s spatiotemporal processing in DiMMPose.

#### 3.1.2. Spatiotemporal Scan and Merge Mechanisms

To achieve this, STMB employs four distinct Spatiotemporal Scan operations, each with a unique scanning function, $\phi_{i}$, designed to associate temporal frames with skeletal structures over the flattened frame-joint skeleton sequence:

(8) $${\tilde{F}}_{l}^{\left(i\right)}=\phi_{i}\left(F_{l}\right),i=1,2,3,4,$$

where ${\tilde{F}}_{l}^{\left(i\right)}\in R^{K\times D}$ denotes the sequence produced by the *i*-th Spatiotemporal Scan order. Each scan order preserves the same frame-joint tokens, but changes their traversal order, allowing PMB to perceive complementary frame-joint dependencies. These spatiotemporally scanned features are fed into PMB to model dependencies under different frame-joint orders, and are then fused through Spatiotemporal Merge:

The four scanning functions $\phi_{1}$–$\phi_{4}$ correspond to two complementary traversal orders, each applied in both forward and reverse directions. For a token indexed by frame *n* and joint *j*, the frame-first order flattens tokens as *k* = (*n* − 1)*J* + *j*, scanning all joints within each frame before proceeding to the next frame; the joint-first order flattens tokens as *k* = (*j* − 1)*N* + *n*, scanning one joint across all frames before proceeding to the next joint. Their reverse counterparts are obtained by reversing the sequence order (*k* → *K* + 1 − *k*), yielding $\phi_{1}$ (frame-first, forward), $\phi_{2}$ (frame-first, reverse), $\phi_{3}$ (joint-first, forward), and $\phi_{4}$ (joint-first, reverse), as illustrated in Figure 2. The two traversal orders capture complementary spatial and temporal dependencies, while the forward and reverse directions enable the SSM to aggregate context from both ends of each sequence. After PMB processing, each output is restored to the original (*n*, *j*) order by the inverse permutation before fusion in Equation (9).

(9) $${\tilde{F}}_{l}=\frac{1}{4}\sum_{i=1}^{4}PMB\left({\tilde{F}}_{l}^{\left(i\right)}\right),$$

where ${\tilde{F}}_{l}\in R^{K\times D}$ is the integrated output after Spatiotemporal Merge and is reshaped for the subsequent layer. This Spatiotemporal Scan and Merge strategy, supported by PMB’s bidirectional state-space modeling, strengthens temporal continuity and joint coordination while maintaining linear-complexity long-range modeling. Since the scan and merge operations are performed on skeleton tokens rather than image patches, they better match the frame-joint structure of 3D human pose sequences.

#### 3.1.3. Complexity Comparison with Transformer-Based Modeling

**Figure 3:**
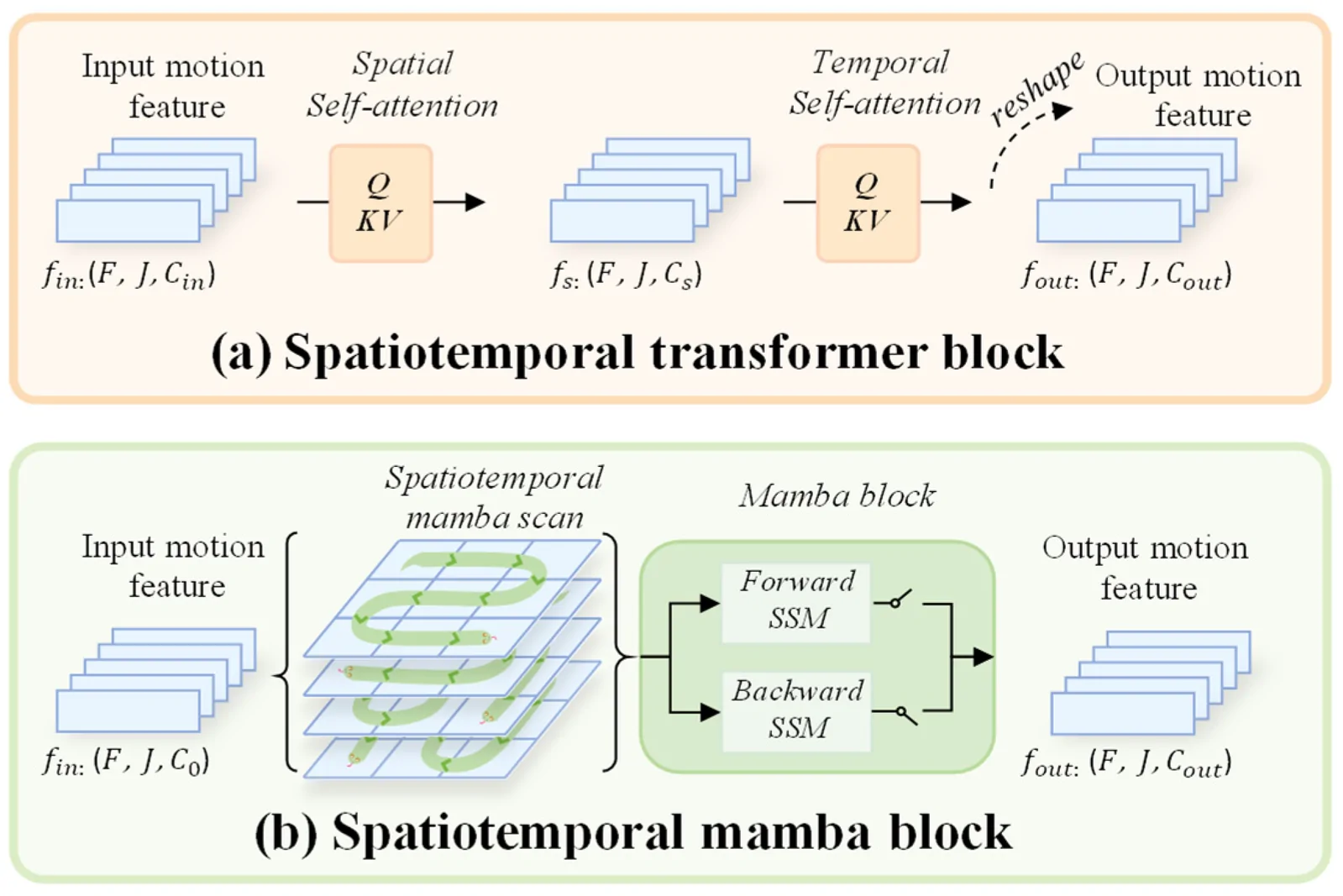
Comparison of representative spatiotemporal modeling architectures. (**a**) Sequential spatial–temporal self-attention; (**b**) the proposed Spatiotemporal Mamba Block using Spatiotemporal Scan and bidirectional state-space propagation.

Figure 3 presents a comparative overview between a representative spatiotemporal Transformer design and the proposed STMB used in DiMMPose. In the example shown in Figure 3a, spatial and temporal dimensions are processed through sequential self-attention operations with corresponding Query-Key-Value (QKV) transformations, followed by reshaping to obtain the output motion features.

In contrast, Figure 3b introduces our proposed spatiotemporal Mamba block (STMB). This block first employs a spatiotemporal Mamba scan to efficiently capture intra-frame-joint dependencies through sequential bidirectional scanning, thereby enhancing the integration of spatial and temporal cues simultaneously. The extracted features subsequently pass through a Mamba block, comprising forward and backward state-space models (SSMs), enabling effective bidirectional propagation and robust spatiotemporal feature aggregation. The resulting state-space representation supports coherent frame-joint feature propagation without relying on quadratic pairwise self-attention. This structural design provides a module-level complexity advantage over Transformer-based spatiotemporal modeling.

Quantitatively, for an input of *N* frames and *J* joints with feature dimension *D*, the sequential spatial–temporal self-attention in Figure 3a incurs *O*(*NJ*^2^*D* + *N*^2^*JD*) cost from the pairwise spatial and temporal interactions, growing quadratically with both *J* and *N*. In contrast, STMB processes *K* = *NJ* tokens as a single sequence through state-space recurrence, whose computational cost scales linearly with the sequence length. The overall cost of STMB is therefore *O*(*NJD*), linear in both *N* and *J*, consistent with the profiled MACs/frame values reported in Section 4.5.2.

### 3.2. Multi-Prompt Mamba Denoiser (MPMD)

The Multi-Prompt Mamba Denoiser (MPMD), highlighted in the blue part of Figure 2, performs the reverse diffusion process to recover clean 3D poses from noisy skeletal features. It transforms $F_{\mathrm{noise}\;}$ and the input 2D skeleton sequence $P_{2D}$ as pose conditions, and injects multi-level prompt guidance through cross-attention. It transforms $F_{\mathrm{noise}\;}\in R^{K\times D}$, where K=N×J, into $F_{\mathrm{denoised}\;}\in R^{N\times J\times D}$.

The prompt branch contains three pre-defined prompt groups: $P_{\mathrm{coarse}}$, $P_{\mathrm{fine}}$, and $P_{\mathrm{motion}}$. $P_{\mathrm{coarse}}$ describes coarse action-level and body-level semantics, such as person, verb, speed, head, trunk, upper limb, and lower limb. $P_{\mathrm{fine}\;}$ describes local anatomical cues, such as neck, shoulder, elbow, hand, knee, foot, and joint. $P_{\mathrm{motion}\;}$ describes motion-status attributes, such as position, orientation, angle, direction, balance, and alignment. These pre-defined prompt words are selected according to the pose context. The action-side cue is carried by the image-to-2D-skeleton/action pipeline that provides $P_{2D}$, rather than being derived from ground-truth 3D poses or evaluation targets. The prompt words are tokenized and embedded through the text encoder to form multi-level text-prompt embeddings:

(10) $$E_{\mathrm{text}\;}=LongCLIP\left[P_{\mathrm{coarse}\;}\left\|P_{\mathrm{fine}\;}\right\|P_{\mathrm{motion}\;}\right],$$

To improve adaptability beyond the fixed prompt vocabulary, learnable prompt embeddings $E_{\mathrm{learn}\;}\in R^{M\times D_{p}}$ are introduced and optimized jointly with the denoising network. As illustrated in Figure 2, $E_{\mathrm{text}\;}$ and $E_{\mathrm{learn}\;}$ are concatenated and injected into the skeletal feature through cross-attention. This design allows the preset prompt words to provide explicit semantic, anatomical, and motion priors, while the learnable embeddings adapt these priors to variations in action categories, body configurations, and motion patterns. The three textual prompt groups are not assigned manually predefined scalar weights; instead, they provide complementary structured conditioning together with the jointly optimized learnable prompt embeddings.

The workflow begins with an initial STMB that processes $F_{\mathrm{noise}\;}$ into ${\tilde{F}}_{l}\in R^{K\times D}$. The prompt-guided cross-attention is formulated as:

(11) $$Q={\tilde{F}}_{l}W_{Q},K_{p}=\left[E_{\mathrm{learn}\;}\|E_{\mathrm{text}\;}\right]W_{K},$$

(12) $$V_{p}=\left[E_{\mathrm{learn}\;}\|E_{\mathrm{text}\;}\right]W_{V},,$$

(13) $${\tilde{F}}^{\prime }=softmax\left(\frac{QK_{p}^{T}}{\sqrt{D}}\right)V_{p}.$$

Here, $Q\in R^{K\times D}$ and $K_{p},V_{p}\in R^{(M+L)\times D}$ are the prompt-attention key and value matrices, respectively. M is the number of learnable prompt tokens, and $L=L_{c}+L_{f}+L_{m}$ is the total number of text-prompt tokens. The prompt-enhanced feature is further refined by stacked STMB layers and an output projection to obtain $F_{\mathrm{denoised}\;}$. In this process, the prompt branch provides semantic and motion priors for denoising, while STMB preserves efficient frame-joint state modeling. The prompt branch does not alter the linear state-space recurrence of STMB, and its practical overhead is evaluated in the efficiency analysis. The textual prompts serve as auxiliary conditioning rather than mandatory pose inputs; when textual cues are removed, STMB and the learnable prompt representations remain available for pose estimation, as evaluated in Section 4.5.4.

## 4. Experiments

### 4.1. Datasets and Evaluation Metrics

Human3.6M [60] serves as a premier indoor benchmark for 3D human pose estimation, featuring an extensive collection of 3.6 million frames captured at a 50 Hz frame rate through a quartet of calibrated cameras. It showcases 11 skilled performers executing 15 varied actions—ranging from ambulatory movements to sedentary tasks and manipulative gestures—each annotated with 3D joint locations using a consistent 17-joint framework. The 15 actions are Directions, Discussion, Eating, Greeting, Phoning, Photo, Posing, Purchases, Sitting, SittingDown, Smoking, Waiting, WalkDog, Walking, and WalkTogether, all of which are included in the evaluation. Recorded in a meticulously controlled studio environment, the dataset offers a detailed portrayal of human kinematics under stable conditions. We adhere to a conventional split, training on subjects S1, S5, S6, S7, and S8, and testing on S9 and S11, fostering equitable comparisons with existing studies. Evaluation hinges on Mean Per Joint Position Error (MPJPE) and Procrustes-aligned MPJPE (P-MPJPE) for spatial precision.

MPI-INF-3DHP [61] broadens the landscape of 3D human pose estimation with a rich assortment of 1.3 million frames spanning diverse settings, from controlled green-screen studios to open indoor spaces and natural outdoor locales. It includes eight participants engaging in eight training activities and seven test activities, captured under varied conditions that introduce challenges like shifting illumination, partial obscurations, and intricate backgrounds. This dataset stands as a robust testbed for evaluating model adaptability. We assess performance using Mean Per Joint Position Error (MPJPE), Percentage of Correct Keypoints (PCK) with a defined threshold, and Area Under the Curve (AUC), offering a holistic view of efficacy across these demanding contexts.

### 4.2. Implementation Details

DiMMPose stacks *L* Spatiotemporal Mamba Blocks (STMBs), each containing one Pose Mamba Block (PMB), while the Multi-Prompt Mamba Denoiser (MPMD) comprises two STMB layers and one prompt cross-attention layer. Following the commonly adopted training protocol in video-based 3D pose lifting [12], we train the model for 120 epochs with a batch size of 4, using AdamW optimization (*β*_1_ = 0.9, *β*_2_ = 0.999, weight decay = 0.1), with an initial learning rate of 6 × 10^−5^ decayed by 0.993 after each epoch. Unless otherwise specified, the default input length is *N* = 243, and the prompt embedding dimension is set to 512. Following prior diffusion-based 3D HPE practice [62], inference uses 20 hypotheses (*H* = 20) and 10 denoising iterations (*M* = 10) with a maximum timestep *T* = 1000. Experiments are conducted on an NVIDIA RTX 4090 GPU with an Intel Core i9-13900K CPU using PyTorch 2.1.

The forward diffusion process adopts a cosine noise schedule with offset *s* = 0.008 and *β* values clipped to [0, 0.999]; the denoiser is trained to predict *x*_0_ directly rather than the added noise. Reverse inference uses Denoising Diffusion Implicit Models (DDIM) sampling with *η* = 1.0. All experiments use a fixed random seed (seed = 1) for Python 3.14.7, NumPy 2.5.1, and PyTorch (including CUDA), with cuDNN deterministic mode enabled; the standard deviations reported in Section 4.5.2 are obtained over repeated stochastic DDIM sampling with the same trained model. The prompt branch contains *M* = 49 learnable prompt tokens and *L* = *L*c + *L*f + *L*m = 28 text-prompt tokens across seven prompt groups (person, verb, speed, head, trunk, upper limb, and lower limb), giving a total of 77 tokens of dimension 512. All learnable prompt vectors are initialized from Ν(0, 0.02^2^) and optimized jointly with the denoising network, while the text encoder is frozen throughout training. The reported checkpoint is the one achieving the lowest MPJPE on the evaluation split, following the common protocol in [12,62].

### 4.3. Performance of DiMMPose

**Figure 4:**
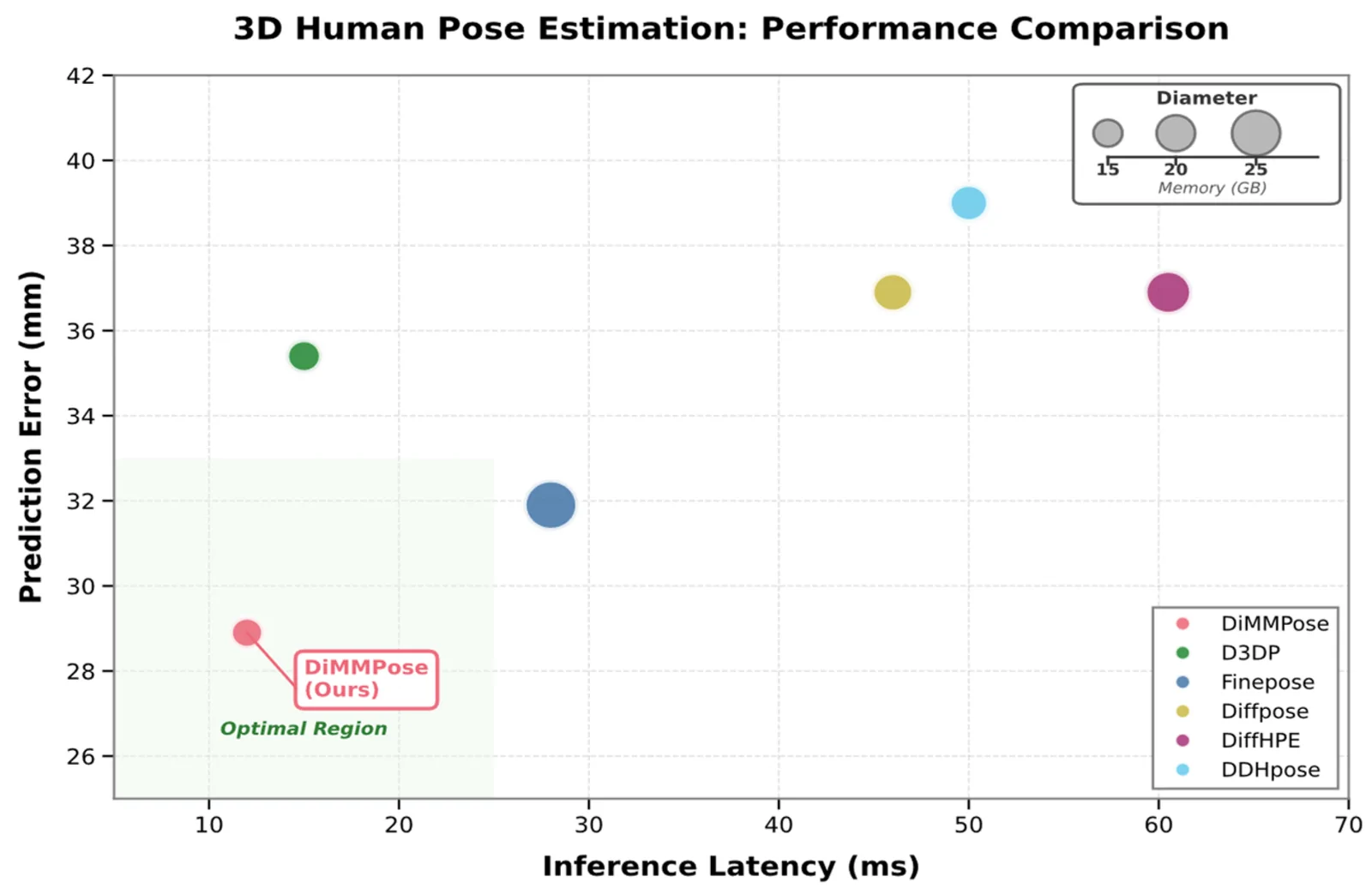
Performance comparison of generative methods for 3D human pose estimation. The x-axis represents inference latency (ms), y-axis denotes Mean Per Joint Position Error (MPJPE, mm), and bubble diameter indicates memory consumption (GB). DiMMPose (red, bottom-left) achieves 28.9 mm error with 12 ms latency and 14.3 GB memory, demonstrating consistent improvements across all metrics compared to existing diffusion-based approaches.

Figure 4 compares the accuracy-efficiency trade-off of diffusion-based 3D HPE methods under the Human3.6M detected 2D input (DET) setting. For a controlled inference-side comparison, Cascaded Pyramid Network (CPN) [63] 2D detections with an input length of *N* = 243 are used, and diffusion inference follows the multi-hypothesis setting with *H* = 20 hypotheses, *M* = 10 denoising iterations, and a maximum timestep of *T* = 1000. Inference latency and peak GPU memory are measured with batch size 1 on an NVIDIA RTX 4090 GPU. The reported latency includes the LongCLIP-based prompt branch after model loading: the predefined prompt words are encoded into text embeddings and then injected through lightweight matrix operations during denoising. Other diffusion baselines, including Diff3DHPE [64], DiffHPE [65], and DDHPose [66], are included in the same accuracy-efficiency comparison. Under this setting, DiMMPose achieves 28.9 mm MPJPE with 12 ms latency and 14.3 GB memory. Compared with FinePOSE [37] (31.9 mm, 28 ms, 23 GB), DiMMPose reduces MPJPE by 3.0 mm, decreases latency by 57.1%, and reduces memory consumption by 37.8%. Compared with D3DP [62] (35.4 mm, 15 ms, 15 GB), it improves MPJPE by 6.5 mm while using slightly lower latency and memory. These results indicate that DiMMPose provides a favorable balance between accuracy and computational cost among diffusion-based pose lifting methods.

### 4.4. 3DHPE on Benchmarks

#### 4.4.1. Evaluation on Human3.6M

We evaluate DiMMPose on Human3.6M under both detected 2D input (DET) and ground-truth 2D input (GT) protocols. Table 1 reports the overall MPJPE and P-MPJPE, and Table 2 presents action-specific MPJPE under the DET setting. With CPN [63] detections and *N* = 243, DiMMPose achieves 28.9 mm MPJPE and 22.5 mm P-MPJPE, improving over FinePOSE [37] by 3.0 mm and 2.5 mm, and over D3DP [62] by 6.5 mm and 6.2 mm, respectively. Table 1 also includes recent regression and state-space baselines, such as MotionBERT [3], PoseMamba [52], and SAMA [54]. Under the GT setting, DiMMPose obtains 15.3 mm MPJPE, giving the best result among the diffusion-based methods listed in Table 1, although the strongest state-space regression baselines perform better with clean GT 2D inputs. This contrast indicates that DiMMPose retains effective 3D lifting capability under clean inputs, while its relative advantage becomes more pronounced in the DET setting, where 2D detection noise and temporal ambiguity make denoising and robust spatiotemporal modeling more important.

**Table 1:** Overall comparison on Human3.6M under detected and ground-truth 2D inputs. Mean Per Joint Position Error (MPJPE) and Procrustes-aligned MPJPE (P-MPJPE) are reported in millimeters. *N* denotes the input sequence length. Red and blue indicate the best and second-best results, respectively. § denotes state-space modeling methods. † denotes reported multi-hypothesis methods. DET denotes detected 2D input. Cascaded Pyramid Network (CPN) [63] detections are used for DET results unless otherwise specified.

Method	Pub.	*N*	Human3.6M (DET)		Human3.6M (GT)
			MPJPE ↓	P-MPJPE ↓	MPJPE ↓
TCN [2]	CVPR’19	–	46.8	36.5	37.8
Anatomy [67]	CSVT’21	–	44.1	35.0	32.3
P-STMO [68]	ECCV’22	243	42.8	34.4	29.3
MixSTE [12]	CVPR’22	243	39.8	30.6	21.6
MHFormer [41]	CVPR’22	351	43.0	34.4	30.5
PoseFormerV2 [17]	CVPR’23	243	45.2	35.6	35.5
DiffPose [13]	CVPR’23	81	36.9	28.7	18.9
GLA-GCN [40]	ICCV’23	243	44.4	34.8	21.0
ActionPrompt [59]	ICME’23	243	41.8	29.5	22.7
MotionBERT [3]	ICCV’23	243	37.5	–	16.9
D3DP † [62]	ICCV’23	243	35.4	28.7	18.4
FinePOSE † [37]	CVPR’24	243	31.9	25.0	16.7
PoseMamba § [52]	AAAI’25	243	37.1	31.5	14.8
SAMA § [54]	ICCV’25	243	36.9	31.3	11.9
DiMMPose † (ours)	–	243	28.9	22.5	15.3

Table 2 shows that DiMMPose achieves the best average MPJPE of 28.9 mm among the listed methods. Compared with FinePOSE [37], it reduces the average error by 3.0 mm, with notable gains on “Sit” (6.0 mm), “Dir.” (4.9 mm), “Wait” (4.9 mm), and “Eat” (3.5 mm). These categories often contain depth ambiguity or complex joint configurations, where frame-joint dependency modeling is especially useful. Compared with recent non-diffusion baselines, including GraphMLP [44], TCPFormer [69], MotionAGFormer [45], and MotionBERT [3], DiMMPose also maintains a lower average error, indicating robust performance across diverse action categories.

**Table 2:** Action-specific MPJPE comparison of DiMMPose on Human3.6M (DET) against SOTA 3D HPE methods.

Method	Pub.	Dir.	Disc.	Eat	Greet	Phone	Photo	Pose	Pur.	Sit	SitD.	Smoke	Wait	WalkD.	Walk	WalkT.	Avg
StridedFormer [20]	TMM’22	40.3	43.3	40.2	42.3	45.6	52.3	41.8	40.5	55.9	60.6	44.2	43.0	44.2	30.0	30.2	43.7
MHFormer [41]	CVPR’22	39.2	43.1	40.1	40.9	44.9	51.2	40.6	41.3	53.5	60.3	43.7	41.1	43.8	29.8	30.6	43.0
P-STMO [68]	ECCV’22	38.9	42.7	40.4	41.1	45.6	49.7	40.9	39.9	55.5	59.4	44.9	42.2	42.7	29.4	29.4	42.8
MixSTE [12]	CVPR’22	37.6	40.9	37.3	39.7	42.3	49.9	40.1	39.8	51.7	55.0	42.1	39.8	41.0	27.9	27.9	40.9
STCFormer [70]	CVPR’23	38.4	41.2	36.8	38.0	42.7	50.5	38.7	38.2	52.5	56.8	41.8	38.4	40.2	26.2	27.7	40.5
GLA-GCN [40]	ICCV’23	41.3	44.3	40.8	41.8	45.9	54.1	42.1	41.5	57.8	62.9	45.0	42.8	45.9	29.4	29.9	44.4
ActionPrompt [59]	ICME’23	37.7	40.2	39.8	40.6	43.1	48.0	38.8	38.9	50.8	63.2	42.0	40.0	42.0	30.5	31.6	41.8
MotionBERT [3]	ICCV’23	36.1	37.5	35.8	32.1	40.3	46.3	36.1	35.3	46.9	53.9	39.5	36.3	35.8	25.1	25.3	37.5
KTPFormer [18]	CVPR’24	37.3	39.2	35.9	37.6	42.5	48.2	38.6	39.0	51.4	55.9	41.6	39.0	40.0	27.0	27.4	40.1
MotionAGFormer [45]	WACV’24	36.4	38.5	36.8	32.9	40.9	48.5	36.6	34.6	51.7	52.8	41.0	36.8	36.6	26.7	27.1	38.4
TCPFormer [69]	AAAI’25	36.4	37.7	35.9	32.6	40.6	47.3	36.7	34.8	47.7	52.3	39.9	36.8	36.6	26.6	26.8	37.9
GraphMLP [44]	PR’25	32.2	38.2	29.3	33.4	33.5	38.1	38.2	31.7	37.3	38.5	34.2	36.1	35.5	28.0	29.3	34.2
D3DP † [62]	ICCV’23	33.0	34.8	31.7	33.1	37.5	43.7	34.8	33.6	45.7	47.8	37.0	35.0	35.0	24.3	24.1	35.4
FinePOSE † [37]	CVPR’24	31.4	31.5	28.8	29.7	34.3	36.5	29.2	30.0	42.0	42.5	33.3	31.9	31.4	22.6	22.7	31.9
DiMMPose † (ours)		26.5	29.5	25.3	26.7	29.7	35.9	26.6	26.7	36.0	39.8	29.0	27.0	32.1	21.2	22.0	28.9

**Note:** Reports MPJPE (mm) across 15 actions using CPN [63] detector (*N* = 243), except MotionBERT (SH). Lower values indicate better accuracy. † denotes multi-hypothesis methods. Red and blue indicate the best and second-best results, respectively.

**Figure 5:**
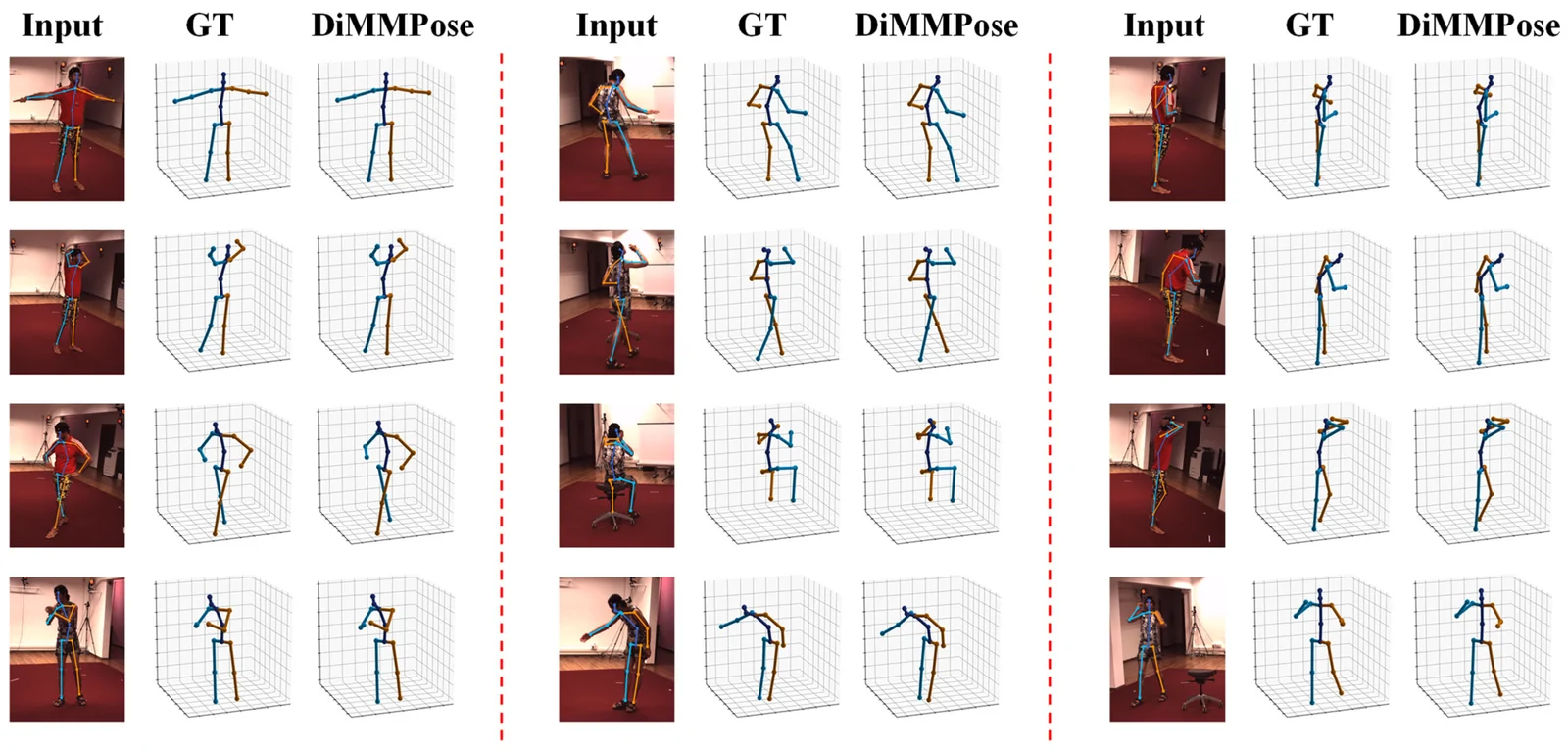
Qualitative results of DiMMPose on Human3.6M. Each group shows the input image with 2D pose overlay, ground-truth 3D skeleton (GT), and DiMMPose prediction. The skeletons are rendered with blue (left side) and orange (right side) to distinguish body parts. Red dashed lines separate different groups of qualitative examples.

Figure 5 presents qualitative results comparing DiMMPose predictions against ground-truth on Human3.6M. For basic actions with extended limbs (Row 1), DiMMPose accurately captures arm positions and body orientation. In challenging scenarios involving self-occlusion and depth ambiguity—such as walking with raised arms (Row 2), seated poses (Row 4), and forward bending (Row 4, right)—DiMMPose consistently reconstructs anatomically plausible skeletons that closely align with ground-truth. These visualizations demonstrate that DiMMPose effectively handles diverse poses with significant viewpoint and depth variations, complementing the quantitative improvements reported in Table 1 and Table 2.

#### 4.4.2. Evaluation on MPI-INF-3DHP

We evaluate DiMMPose on MPI-INF-3DHP to assess its performance under more diverse indoor and outdoor conditions. As shown in Table 3, prior methods are reported with different input sequence lengths, so we list *N* for each method and report DiMMPose under both *N* = 81 and *N* = 243. With *N* = 81, DiMMPose achieves 98.6% PCK, 81.2 AUC, and 24.9 mm MPJPE, showing competitive performance without relying on a longer input sequence. With *N* = 243, DiMMPose further improves to 99.0% PCK, 84.1 AUC, and 23.0 mm MPJPE. Compared with 243-frame diffusion-based baselines, DiMMPose reduces MPJPE by 3.2 mm over FinePOSE [37] and by 5.1 mm over D3DP [62], while improving AUC by 4.1 and 5.0, respectively. These results suggest that the proposed spatiotemporal denoising design benefits pose estimation under diverse scene conditions, especially when longer temporal context is available.

**Figure 6:**
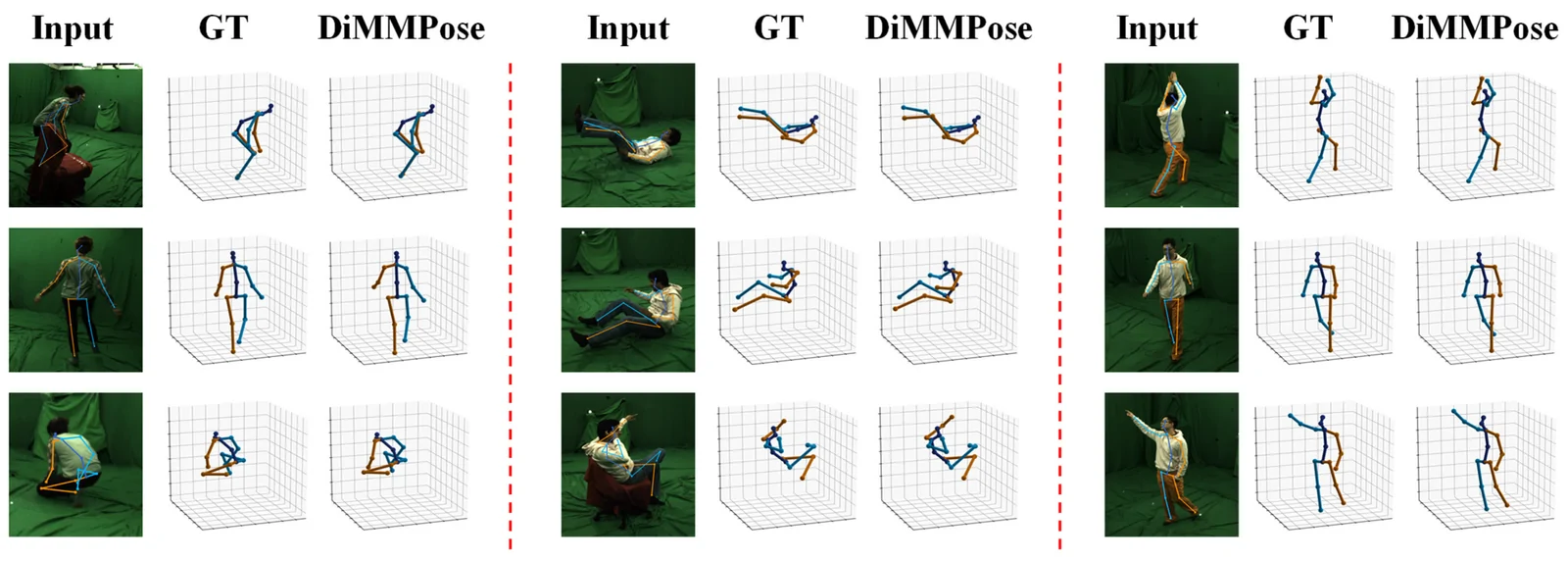
Qualitative results of DiMMPose on MPI-INF-3DHP. Each group displays the input image with 2D pose overlay, ground-truth 3D skeleton (GT), and DiMMPose prediction. The results demonstrate robust performance across challenging scenarios including lying, crouching, and seated poses under varied environmental conditions. Blue and orange denote the left and right body sides, respectively; red dashed lines separate different groups of qualitative examples.

Figure 6 further illustrates DiMMPose predictions against ground-truth on MPI-INF-3DHP across diverse scenarios. The dataset presents additional challenges including green screen backgrounds, varied lighting conditions, and unconventional poses such as lying (Row 2, middle), crouching (Row 3, left), and seated positions (Row 2–3, middle). Despite these complexities, DiMMPose consistently produces accurate 3D reconstructions that closely match the ground-truth skeletons. Notably, for poses with extreme depth variations—such as the lying and reaching poses—DiMMPose successfully recovers correct limb orientations and joint positions, illustrating robust behavior under diverse pose and environmental conditions beyond the controlled Human3.6M setting.

**Figure 7:**
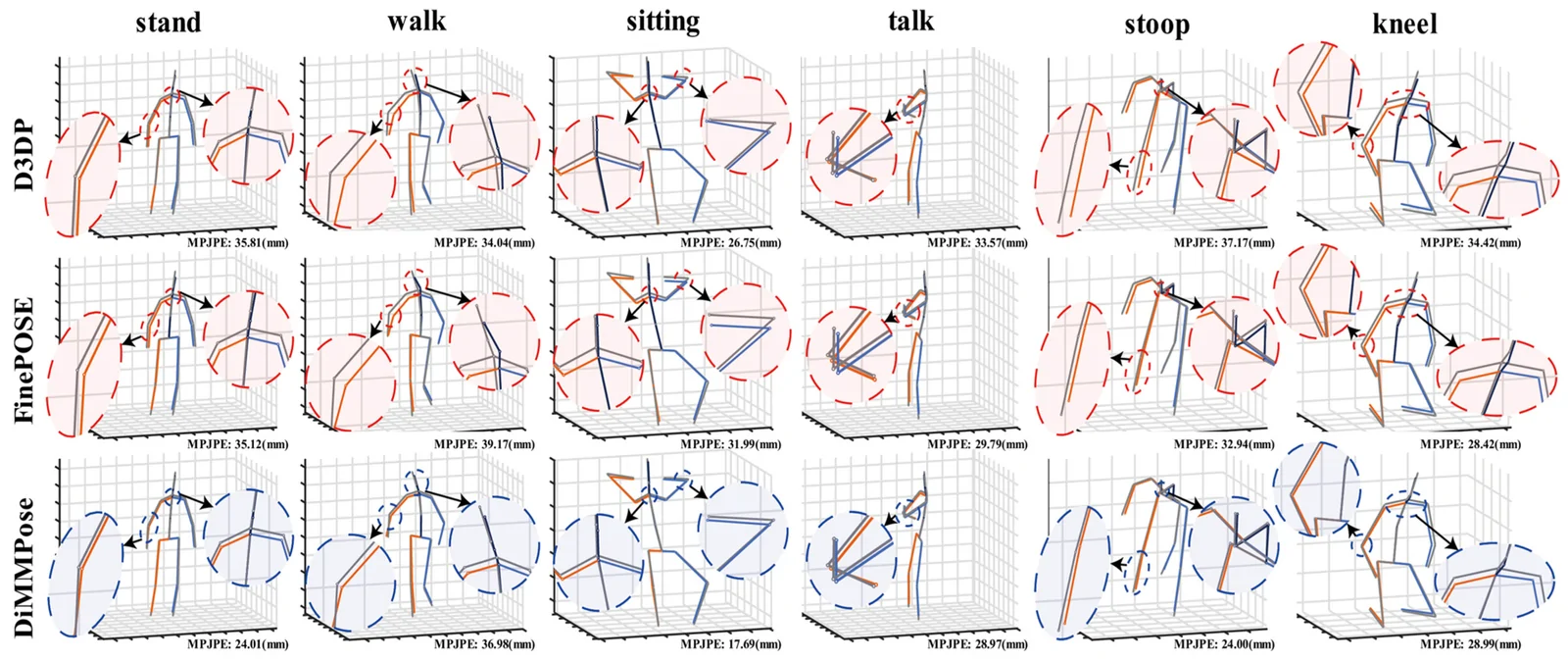
Qualitative comparison of D3DP, FinePOSE, and DiMMPose on MPI-INF-3DHP. The gray skeleton denotes the ground-truth 3D pose. Predicted poses are shown in blue (left side) and orange (right side). MPJPE (mm) is reported below each pose example as the corresponding 3D joint-position error. Red and blue dashed annotations highlight visually noticeable local differences for the compared methods and DiMMPose, respectively; they do not represent an additional joint-angle metric.

Figure 7 visually compares D3DP, FinePOSE, and DiMMPose on MPI-INF-3DHP. The per-example MPJPE values quantify 3D joint-position error, while the dashed annotations only highlight local visual differences. For simpler actions (“Stand,” “Walk”), DiMMPose produces 3D poses that are visually closer to the ground-truth than D3DP and FinePOSE, especially around challenging regions such as the right arm, shoulder, and head. For complex motions (“Talk,” “Stoop,” and “Kneel”), DiMMPose better preserves limb orientation and body structure in the highlighted regions. These results provide qualitative evidence consistent with the quantitative improvements reported in Table 3.

### 4.5. Ablation Studies

#### 4.5.1. Impact of Multi-Prompt and STMB Strategies

The following ablations evaluate the main architectural factors introduced in DiMMPose while keeping the diffusion inference configuration fixed according to Section 4.2. We evaluate the individual and combined effects of Multi-Prompt guidance and STMB on Human3.6M (DET), as detailed in Table 4. Starting from the FinePOSE baseline (a), which obtains 31.9 mm MPJPE and 25.0 mm P-MPJPE, adding Multi-Prompt guidance (b) reduces MPJPE to 31.2 mm (↓0.7 mm) and P-MPJPE to 24.5 mm (↓0.5 mm), indicating that prompt-guided denoising provides useful contextual regularization. Replacing the baseline spatiotemporal module with STMB (c) further decreases MPJPE to 30.0 mm (↓1.9 mm) and P-MPJPE to 23.2 mm (↓1.8 mm), improving long-range frame-joint dependency modeling. DiMMPose (d), combining both strategies, achieves 28.9 mm MPJPE and 22.5 mm P-MPJPE, surpassing (b) by 2.3 mm and 2.0 mm, and (c) by 1.1 mm and 0.7 mm, respectively, showing that prompt-guided denoising and Mamba-based spatiotemporal modeling provide complementary gains.

#### 4.5.2. Efficiency of STMB Configuration

Table 5 evaluates the performance and efficiency of 1-way, 2-way, and 4-way STMB variants based on the Spatiotemporal Scan and Merge mechanism on Human3.6M (DET). The 1-way variant obtains 32.3 ± 0.5 mm MPJPE and 25.5 ± 0.5 mm P-MPJPE, indicating limited spatiotemporal integration with a single scan direction. Expanding to 2-way scanning reduces MPJPE to 30.9 ± 0.5 mm (↓1.4 mm) and P-MPJPE to 24.3 ± 0.4 mm (↓1.2 mm), showing that complementary scan orders provide richer frame-joint dependency coverage than a single traversal order. The 4-way variant achieves the best accuracy, further reducing MPJPE/P-MPJPE by 0.9/0.6 mm to 30.0 ± 0.3/23.7 ± 0.5 mm. Notably, all variants keep the same MACs/frame and parameter size (14.66 and 0.8 MB), because the additional scan directions operate on the same feature representation, and the Merge operation does not introduce extra learnable branches. These results suggest that richer scan directions improve spatiotemporal feature coverage while preserving the profiled computational cost; therefore, we adopt the 4-way variant as the default STMB configuration. PMB bidirectionality and multi-directional Scan-and-Merge serve different roles: bidirectional propagation incorporates preceding and following context within each scanned sequence, whereas multiple scan directions provide complementary context across different frame-joint traversal orders.

#### 4.5.3. Visualization of STMB Effectiveness

**Figure 8:**
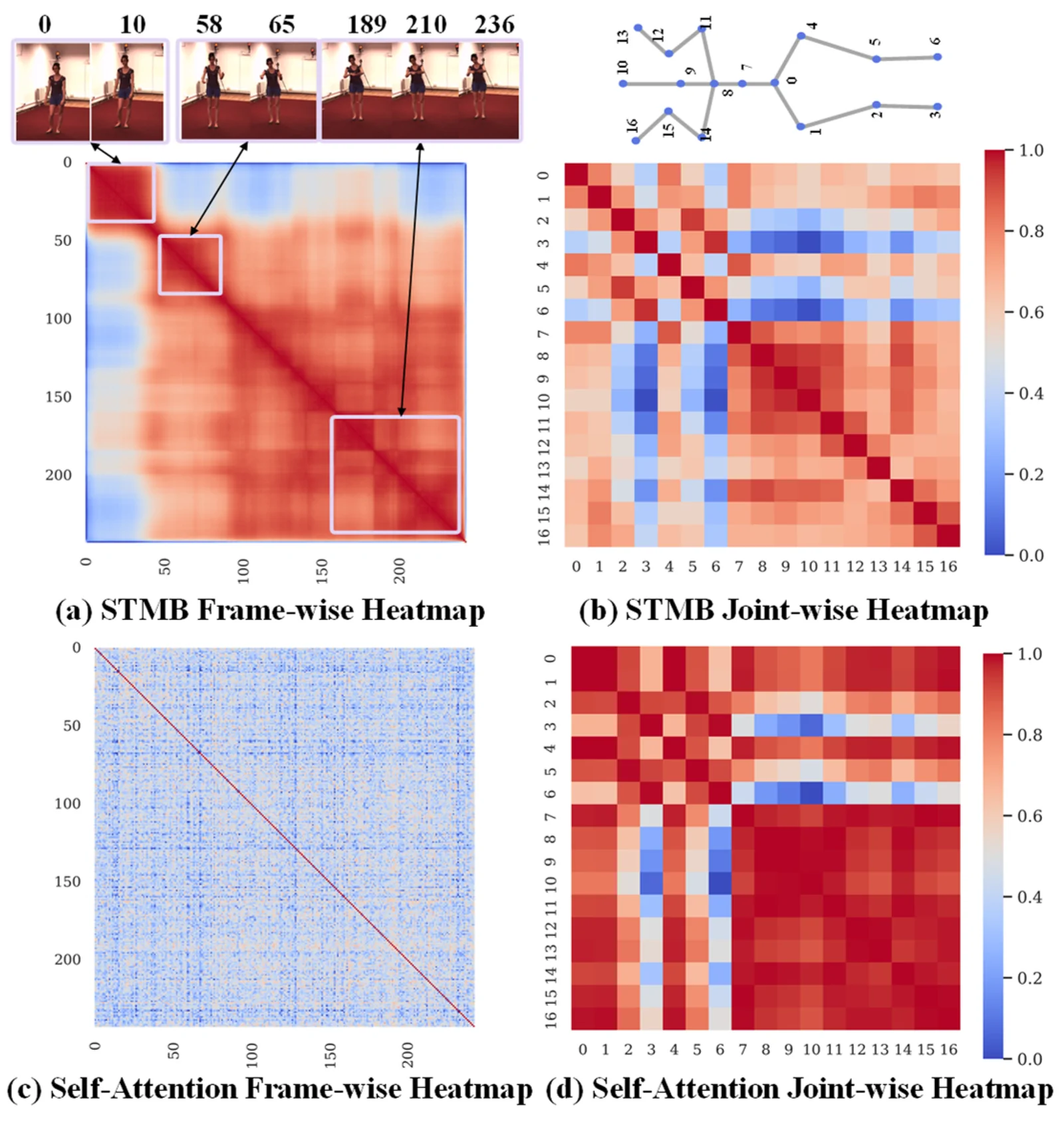
Heatmaps comparing STMB and Self-Attention on Frame and Joint Correlations. Random sample, *F* = 243, *J* = 17, normalized. Red indicates high correlation, blue indicates low. (**a**) STMB Frame-wise, (**b**) STMB Joint-wise (65th frame), (**c**) Self-Attention Frame-wise, (**d**) Self-Attention Joint-wise (65th frame).

We evaluate STMB’s impact on skeletal sequence features in DiMMPose using heatmaps on Human3.6M (DET), comparing STMB and Self-Attention correlations in Figure 8. Here, motion coherence refers to the temporal consistency of skeletal representations across consecutive and long-range frames rather than to a separately defined evaluation metric. The STMB Frame-wise heatmap (a) shows clearer temporal correlations at frames 0–10 (hands down), 58–65 (hands up), and 189–236 (hands crossed), indicating stronger long-range temporal associations. In contrast, the Self-Attention Frame-wise heatmap (c) shows a uniform distribution, indicating weaker long-range temporal associations. For spatial modeling, STMB Joint-wise (b) and Self-Attention Joint-wise (d) heatmaps at the 65th frame both exhibit high joint dependencies, indicating comparable spatial correlation patterns. These observations support STMB’s ability to capture long-range spatiotemporal dependencies; the clearer temporal correlations are consistent with the bidirectional contextual propagation described in Equations (3)–(7), while Table 5 further shows that complementary scan directions improve pose-estimation accuracy.

#### 4.5.4. Effect of Prompt Types in MPMD

We analyze the impact of prompt types in the MPMD framework on DiMMPose’s performance using Human3.6M (DET), as shown in Table 6. The baseline DiMMPose achieves an MPJPE of 28.9 mm and P-MPJPE of 22.5 mm. Excluding text prompts (w/o $\left.P_{\mathrm{text}\;}\right)$ raises MPJPE to 30.0 mm (+1.1 mm) and P-MPJPE to 23.7 mm (+1.2 mm), indicating their contribution to contextual refinement. Ablating coarse prompts (w/o $P_{\mathrm{coarse}\;}$) slightly affects accuracy, with MPJPE at 29.1 mm (+0.2 mm) and P-MPJPE at 23.0 mm (+0.5 mm). Removing fine prompts (w/o $P_{\mathrm{fine}\;}$) results in 29.5 mm MPJPE (+0.6 mm) and 23.2 mm P-MPJPE (+0.7 mm), showing localized improvements. Excluding motion prompts (w/o $P_{\mathrm{motion}\;}$) yields 29.9 mm MPJPE (+1.0 mm) and 23.7 mm P-MPJPE (+1.2 mm), showing that motion-status cues provide important temporal guidance during denoising. Removing learnable embeddings (w/o $E_{\mathrm{learn}\;}$) increases MPJPE to 29.6 mm (+0.7 mm) and P-MPJPE to 23.5 mm (+1.0 mm), confirming their adaptability. Overall, these results show that MPMD benefits from both structured textual prompts and learnable prompt adaptation, with motion-status and fine-grained cues contributing most to the observed gains. These ablation differences quantify the contribution of each prompt component rather than predefined scalar weights. No fixed manual weights are assigned to the three textual prompt groups; instead, the structured textual prompts are combined with jointly optimized learnable prompt representations.

**Figure 9:**
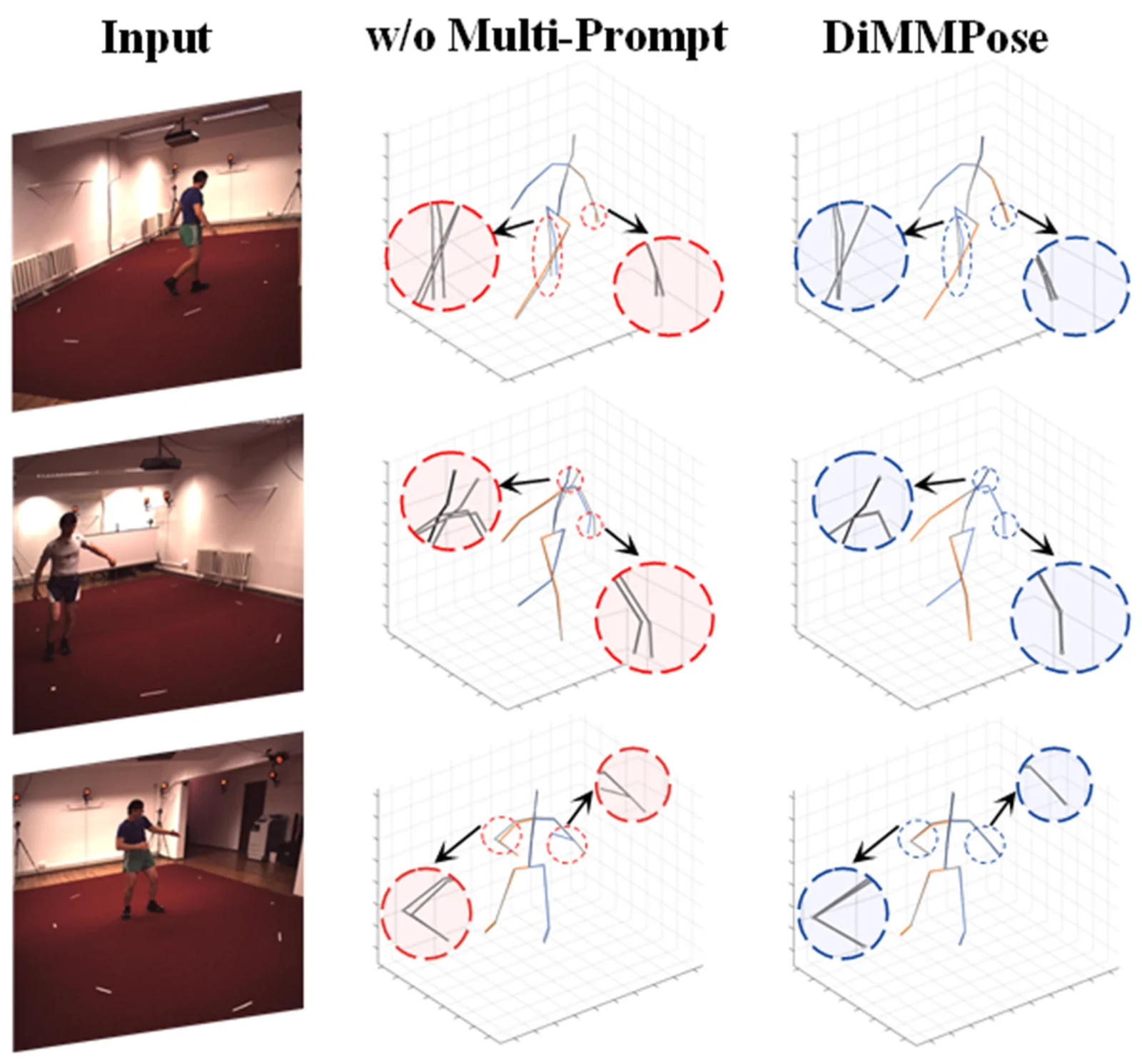
Qualitative comparison of multi-prompt guidance effectiveness. Each row displays: (**left**) input RGB image, (**middle**) predicted 3D pose without multi-prompt guidance showing localization errors (red circles), (**right**) prediction from full DiMMPose demonstrating improved accuracy (blue circles).

Figure 9 presents qualitative comparisons across three representative test cases from Human3.6M. The middle column shows predictions without multi-prompt guidance, while the right column displays results from the full DiMMPose framework. Without multi-prompt guidance, the model produces noticeable joint-localization errors in anatomically constrained regions such as elbows, knees, and torso connections (red circles), resulting in visually less plausible local configurations. In contrast, the full DiMMPose produces improved joint localization and body structure in these regions (blue circles). These visual differences are consistent with the quantitative gains reported in Table 6 and suggest that structured multi-level prompts provide useful anatomical and motion-related conditioning, particularly when 2D detections are noisy or ambiguous.

### 4.6. Robustness Analysis Under Occlusion

**Figure 10:**
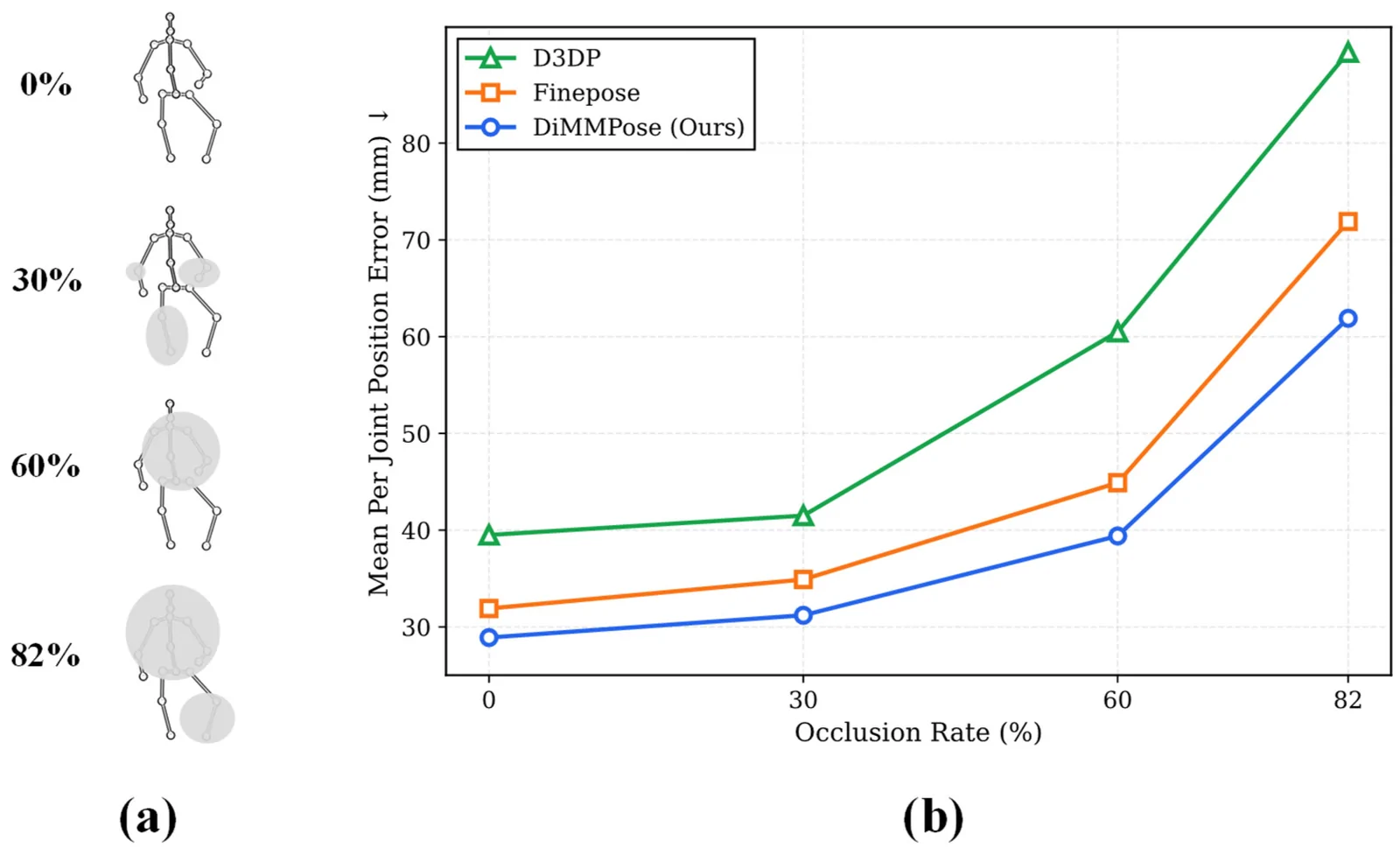
Robustness evaluation under synthetic occlusion. (**a**) Occlusion patterns at 0%, 30%, 60%, and 82% (gray regions denote occluded parts). (**b**) MPJPE comparison across occlusion rates.

In this part, we evaluate three diffusion-based methods under synthetic occlusion conditions at four severity levels (0%, 30%, 60%, and 82%), as shown in Figure 10a. Figure 10b presents the MPJPE comparison across occlusion rates. DiMMPose achieves errors of 28.9 mm, 31.2 mm, 39.4 mm, and 61.9 mm at the four occlusion levels, compared to 31.9 mm, 34.9 mm, 44.9 mm, and 71.9 mm for FinePOSE, and 39.5 mm, 41.5 mm, 49.5 mm, and 78.4 mm for D3DP, respectively. These results suggest that DiMMPose maintains relatively stable performance under occlusion, with relative average MPJPE reductions of 12.1% and 22.7% over FinePOSE and D3DP, respectively. The observed robustness is consistent with two design choices. First, MPMD provides multi-level textual conditioning when 2D detections are noisy or incomplete. Second, STMB enables linear-complexity O(*N*) spatiotemporal aggregation through state-space propagation across frame-joint sequences, facilitating the use of contextual information under partial observations. Together, these mechanisms provide complementary support for denoising and spatiotemporal modeling under degraded 2D observations. This controlled synthetic occlusion evaluation is complemented by the MPI-INF-3DHP results in Section 4.4.2, which assess performance under more diverse indoor/outdoor conditions with partial obscurations, illumination variation, and complex backgrounds.

### 4.7. Long-Sequence Memory Usage

**Figure 11:**
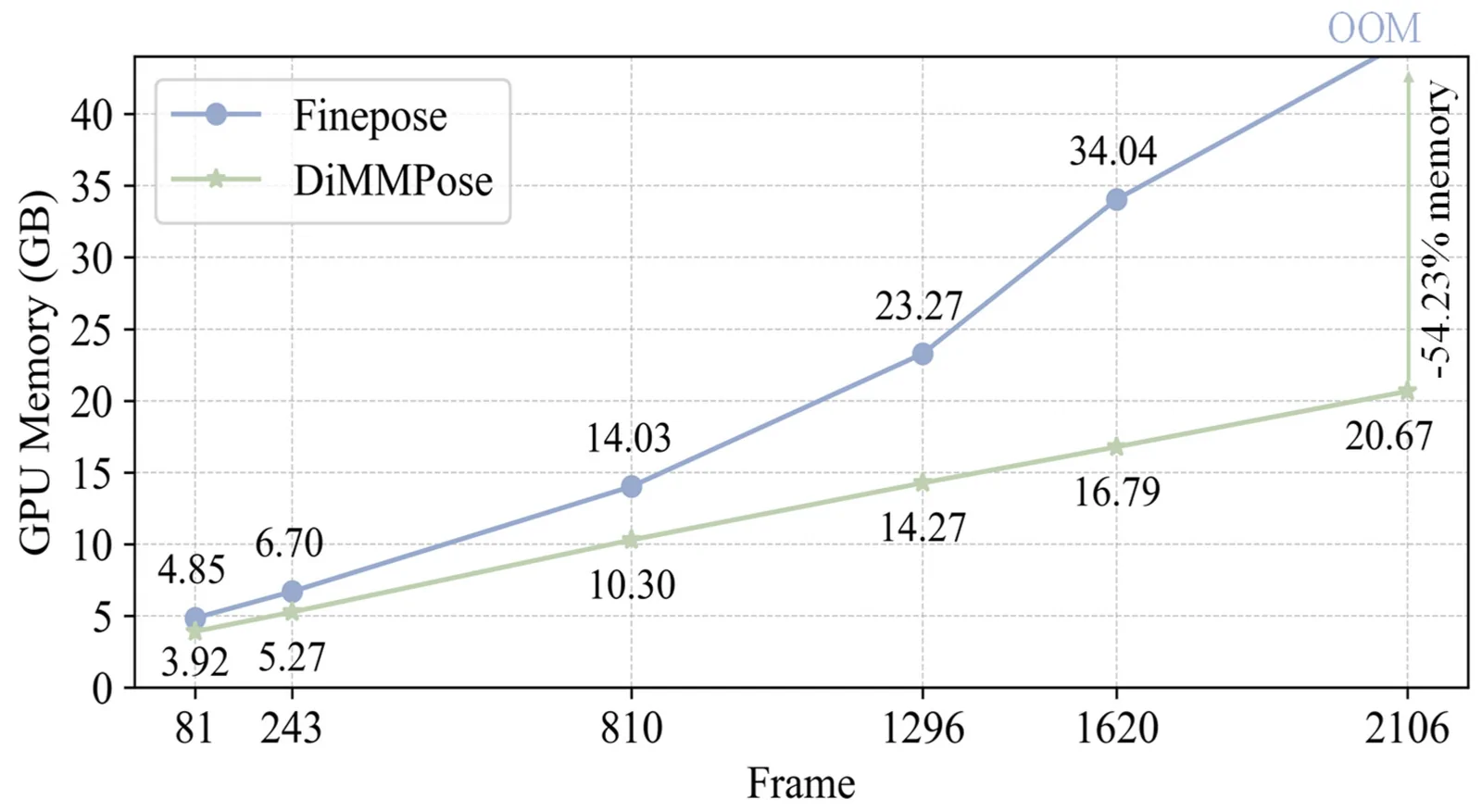
GPU memory consumption of frame length for DiMMPose and FinePOSE. Note: measured on NVIDIA RTX 4090 with batch size 1. Lower memory usage indicates better efficiency. OOM (Out of Memory) observed for FinePOSE at high frames.

As shown in Figure 11, we evaluate memory scalability on Human3.6M (DET) with batch size 1 by increasing the input length from 81 to 2106 frames. This experiment is intended as a long-sequence memory stress test rather than evidence that longer inputs necessarily improve MPJPE, P-MPJPE, PCK, or AUC. At the default *N* = 243 setting used in the main benchmark, DiMMPose reduces peak GPU memory from 6.70 GB to 5.27 GB, corresponding to a 21.3% reduction. The gap becomes more pronounced as the sequence length increases. At 810, 1296, and 1620 frames, DiMMPose uses 10.30, 14.27, and 16.79 GB, compared with 14.03, 23.27, and 34.04 GB for FinePOSE. When the input length increases to 2106 frames, FinePOSE runs out of memory, whereas DiMMPose remains executable with 20.67 GB. These results indicate that the proposed STMB-based design has better memory scalability and shows potential for long-context 3D pose inference in extended videos. Prediction accuracy is evaluated up to *N* = 243 in Table 3, where increasing the context from *N* = 81 to *N* = 243 improves MPJPE from 24.9 mm to 23.0 mm on MPI-INF-3DHP. The effect of ultra-long inputs beyond this range on pose accuracy is not investigated in this study. Accordingly, Figure 11 demonstrates the computational feasibility of substantially longer-context inference rather than an additional accuracy gain from ultra-long sequences.

### 4.8. Qualitative Evaluation on In-the-Wild Videos

**Figure 12:**
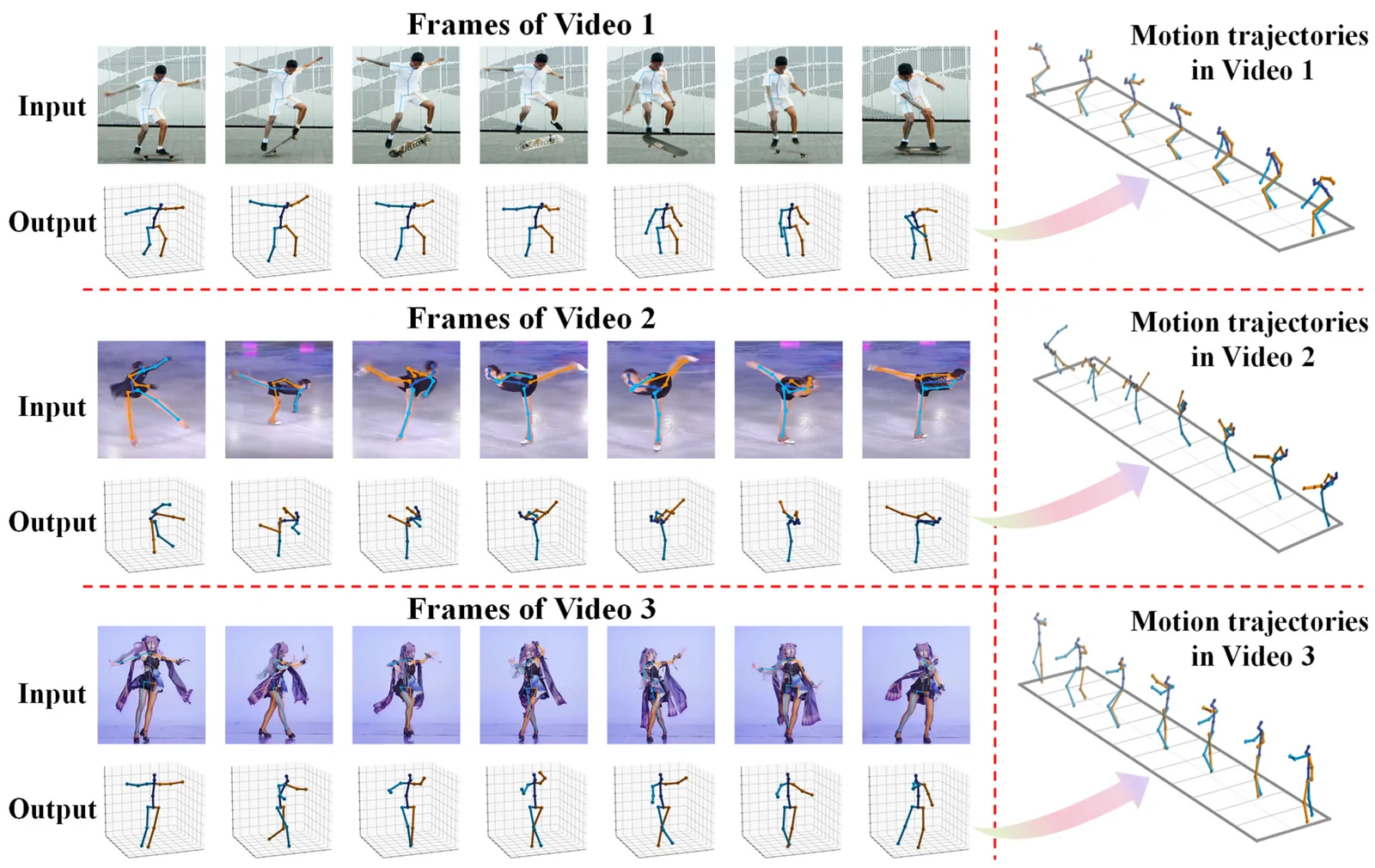
Qualitative results on in-the-wild videos. Three video sequences are presented: skateboarding (Video 1), figure skating (Video 2), and animated character dancing (Video 3). Left: input frames with 2D pose overlays and corresponding 3D pose predictions. Right: aggregated predicted motion trajectories showing the temporal evolution of the reconstructed poses. Ground-truth 3D annotations are unavailable for these sequences; therefore, the trajectories are used for qualitative visualization rather than quantitative error evaluation. Red dashed lines separate the three video sequences; arrows indicate the mapping from frame-wise 3D predictions to the aggregated motion trajectories; blue and orange denote the two body sides.

To qualitatively examine the behavior of DiMMPose beyond controlled benchmarks, we test the full RGB-to-3D pose lifting pipeline on in-the-wild videos from diverse scenarios. For each video, HRNet [71] is first used to estimate 2D human skeletons from RGB frames, and DiMMPose then lifts the detected 2D poses into 3D pose sequences. No domain-specific fine-tuning is applied. As shown in Figure 12, the pipeline produces visually consistent 3D pose sequences for skateboarding with rapid body rotations and dynamic balance changes (Video 1), figure skating with fast spinning motions and large limb extensions (Video 2), and animated character dancing with non-realistic appearance and complex choreography (Video 3). The trajectory visualizations further illustrate smooth evolution of the predicted poses across frames, showing that DiMMPose can operate with practical 2D detector outputs and suggesting potential applications in sports analysis, entertainment, and motion capture for animation. Since ground-truth 3D annotations are unavailable for these videos, Figure 12 is used only as qualitative in-the-wild evidence rather than as a quantitative generalization or 3D accuracy evaluation.

## 5. Conclusions

This work introduces DiMMPose, a diffusion-based framework for 3D human pose estimation, leveraging Mamba’s state-space model to address computational costs, weak long-range joint connections, and limited supervision signals. DiMMPose integrates three key components: the Pose Mamba Block (PMB) for efficient long-range modeling, the Spatiotemporal Mamba Block (STMB) for unified frame-joint dependencies, and the Multi-Prompt Mamba Denoiser (MPMD) for structured prompt-guided denoising. Experiments on Human3.6M and MPI-INF-3DHP demonstrate that DiMMPose achieves 28.9 mm MPJPE on Human3.6M (DET) and 23.0 mm MPJPE on MPI-INF-3DHP (*N* = 243), surpassing FinePOSE by 3.0 mm and 3.2 mm, respectively. In memory scalability tests, DiMMPose reduces peak GPU memory by 21.3% at the default *N* = 243 setting and remains executable at 2106 frames, where FinePOSE runs out of memory, indicating its potential for long-context pose inference. The visualization analysis further illustrates STMB’s ability to capture spatiotemporal correlations, complementing the quantitative accuracy and efficiency results.

## Figures and Tables

**Table 3:** PCK, AUC, and MPJPE comparison of DiMMPose on MPI-INF-3DHP against SOTA 3D HPE methods.

Method	*N*	MPI-INF-3DHP			Year
		PCK ↑	AUC ↑	MPJPE ↓	
TCN [2]	81	86	51.9	84.0	CVPR’19
Anatomy [67]	81	87.9	54	78.8	CSVT’21
P-STMO [68]	81	97.9	75.8	32.2	ECCV’22
MixSTE [12]	27	94.4	66.5	54.9	CVPR’22
PoseFormerV2 [17]	81	97.9	78.8	27.8	CVPR’23
MHFormer [41]	9	93.8	63.3	58.0	CVPR’22
GLA-GCN [40]	81	98.5	79.1	27.8	ICCV’23
KTPFormer [18]	81	98.9	83.9	23.1	CVPR’24
Diffpose [23]	81	98.0	75.9	29.1	CVPR’23
D3DP [62]	243	98.0	79.1	28.1	ICCV’23
FinePOSE [37]	243	98.9	80.0	26.2	CVPR’24
UDM-HPE [22]	243	99.2	80.2	25.9	Neuro’25
DiMMPose (ours)	81	98.6	81.2	24.9	
	243	99.0	84.1	23.0	

**Note:** Reports PCK (%), AUC, and MPJPE (mm) with varying frame lengths (*N*). Higher PCK/AUC and lower MPJPE indicate better accuracy. Red and blue indicate the best and second-best results, respectively. Gray shading denotes DiMMPose (ours).

**Table 4:** Ablation study on the impact of Multi-Prompt and STMB strategies on DiMMPose’s MPJPE and P-MPJPE on Human3.6M (DET). Reports MPJPE and P-MPJPE (mm) for FinePOSE baseline (a), with Multi-Prompt (b), STMB (c), and DiMMPose (d) combining both. Lower values indicate better accuracy. ✓ indicates that the corresponding component is included. ↓ indicates that a lower value is better. Red and blue indicate the best and second-best results, respectively.

Component/Metric	(a)	(b)	(c)	(d)
Baseline	**✓**	**✓**	**✓**	**✓**
w/Multi-Prompt		**✓**		**✓**
w/STMB			**✓**	**✓**
MPJPE ↓	31.9	31.2	30.0	28.9
P-MPJPE ↓	25.0	24.5	23.2	22.5

**Table 5:** Performance and efficiency analysis of STMB Variants on DiMMPose’s MPJPE and P-MPJPE on Human3.6M (DET). Reports MPJPE and P-MPJPE (mm), MACs/frame (M), and Param (MB) of a single STMB module for 1-way, 2-way, and 4-way configurations. All three variants retain the same *K* = 4 SSM parameter groups; the 1-way and 2-way settings restrict traversal diversity rather than the parameterization, so the MACs/frame and parameter size are identical by construction. Values include standard deviation over repeated stochastic sampling. Lower MPJPE/P-MPJPE indicate better accuracy.

STMB Variant	MPJPE ↓	P-MPJPE ↓	MACs/Frame (M)	Param. (MB)
1-way	32.3 ± 0.5	25.5 ± 0.5	14.66	0.8
2-way	30.9 ± 0.5	24.3 ± 0.4	14.66	0.8
4-way	30.0 ± 0.3	23.7 ± 0.5	14.66	0.8

**Table 6:** Ablation study on prompt types in MPMD framework: performance impact on DiMMPose’s MPJPE and P-MPJPE on Human3.6M (DET). Presents MPJPE and P-MPJPE (mm) with DiMMPose baseline (28.9 mm, 22.5 mm). Values in parentheses show error increase (mm) relative to baseline. Lower values indicate better accuracy. Red values in parentheses indicate the error increase relative to the full DiMMPose baseline.

Method	Human3.6M (DET)	
	MPJPE ↓	P-MPJPE ↓
DiMMPose	28.9	22.5
w/o $P_{\mathrm{text}\;}$	30.0 (+1.1)	23.7 (+1.2)
w/o $P_{\mathrm{coarse}\;}$	29.1 (+0.2)	23.0 (+0.5)
w/o $P_{\mathrm{fine}\;}$	29.5 (+0.6)	23.2 (+0.7)
w/o $P_{\mathrm{motion}\;}$	29.9 (+1.0)	23.7 (+1.2)
w/o $E_{\mathrm{learn}\;}$	29.6 (+0.7)	23.5 (+1.0)

## Data Availability

The datasets used in this study (Human3.6M and MPI-INF-3DHP) are available from their original providers. The code will be made publicly available at https://github.com/ocean-xuli/DiMMPose (accessed on 27 August 2026) after publication.

## References

[1] Li S., He X., Song W., Hao A., Qin H. (2023). Graph diffusion convolutional network for skeleton based semantic recognition of two-person actions. IEEE Trans. Pattern Anal. Mach. Intell..

[2] Pavllo D., Feichtenhofer C., Grangier D., Auli M. 3d human pose estimation in video with temporal convolutions and semi-supervised training. Proceedings of the IEEE/CVF Conference on Computer Vision and Pattern Recognition.

[3] Zhu W., Ma X., Liu Z., Liu L., Wu W., Wang Y. Motionbert: A unified perspective on learning human motion representations. Proceedings of the IEEE/CVF International Conference on Computer Vision.

[4] Liu J., Rojas J., Li Y., Liang Z., Guan Y., Xi N., Zhu H. A graph attention spatio-temporal convolutional network for 3D human pose estimation in video. Proceedings of the 2021 IEEE International Conference on Robotics and Automation (ICRA), IEEE.

[5] Saini N., Bonetto E., Price E., Ahmad A., Black M.J. (2022). Airpose: Multi-view fusion network for aerial 3d human pose and shape estimation. IEEE Robot. Autom. Lett..

[6] Giulietti N., Todesca D., Carnevale M., Giberti H. (2025). A Real-Time Human Pose Measurement System for Human-In-The-Loop Dynamic Simulators. IEEE Access.

[7] Wang Y., Liu P., Kang H., Wu D., Miao D. (2025). ICFNet: Interactive-complementary fusion network for monocular 3D human pose estimation. Neurocomputing.

[8] Xue Y., Chen J., Zhang Y., Yu C., Ma H., Ma H. (2022). 3D Human Mesh Reconstruction by Learning to Sample Joint Adaptive Tokens for Transformers. Proceedings of the 30th ACM International Conference on Multimedia, Association for Computing Machinery, Lisboa, Portugal, 10–14 October 2022.

[9] Li Z., Xu B., Huang H., Lu C., Guo Y. Deep Two-Stream Video Inference for Human Body Pose and Shape Estimation. Proceedings of the IEEE/CVF Winter Conference on Applications of Computer Vision (WACV).

[10] Lin K., Wang L., Liu Z. End-to-End Human Pose and Mesh Reconstruction with Transformers. Proceedings of the IEEE/CVF Conference on Computer Vision and Pattern Recognition (CVPR).

[11] Lin K., Wang L., Liu Z. Mesh Graphormer. Proceedings of the IEEE/CVF International Conference on Computer Vision (ICCV).

[12] Zhang J., Tu Z., Yang J., Chen Y., Yuan J. Mixste: Seq2seq mixed spatio-temporal encoder for 3d human pose estimation in video. Proceedings of the IEEE/CVF Conference on Computer Vision and Pattern Recognition.

[13] Gong J., Foo L.G., Fan Z., Ke Q., Rahmani H., Liu J. Diffpose: Toward more reliable 3d pose estimation. Proceedings of the IEEE/CVF Conference on Computer Vision and Pattern Recognition.

[14] Chen Z., Dai J., Pan J., Zhou F. (2025). Diffusion model with temporal constraint for 3D human pose estimation. Vis. Comput..

[15] Cheng Y., Wang B., Yang B., Tan R.T. (2021). Graph and temporal convolutional networks for 3d multi-person pose estimation in monocular videos. Proc. AAAI Conf. Artif. Intell..

[16] Cheng Y., Yang B., Wang B., Tan R.T. 3d human pose estimation using spatio-temporal networks with explicit occlusion training. Proceedings of the AAAI Conference on Artificial Intelligence.

[17] Zhao Q., Zheng C., Liu M., Wang P., Chen C. Poseformerv2: Exploring frequency domain for efficient and robust 3d human pose estimation. Proceedings of the IEEE/CVF Conference on Computer Vision and Pattern Recognition.

[18] Peng J., Zhou Y., Mok P.Y. Ktpformer: Kinematics and trajectory prior knowledge-enhanced transformer for 3d human pose estimation. Proceedings of the IEEE/CVF Conference on Computer Vision and Pattern Recognition.

[19] Zheng S., Cao W. (2025). EHGFormer: An efficient hypergraph-injected transformer for 3D human pose estimation. Image Vis. Comput..

[20] Li W., Liu H., Ding R., Liu M., Wang P., Yang W. (2022). Exploiting temporal contexts with strided transformer for 3d human pose estimation. IEEE Trans. Multimed..

[21] Wei M., Xie X., Zhong Y., Shi G. (2025). Learning Pyramid-structured Long-range Dependencies for 3D Human Pose Estimation. IEEE Trans. Multimed..

[22] Liu Z., Wang Y. (2025). Uncertainty-guided Diffusion Model for 3D Human Pose Estimation. Neurocomputing.

[23] Feng R., Gao Y., Tse T.H.E., Ma X., Chang H.J. Diffpose: Spatiotemporal diffusion model for video-based human pose estimation. Proceedings of the IEEE/CVF International Conference on Computer Vision.

[24] Wan X., Chen Z., Duan B., Zhao X. (2025). Dual-Diffusion for Binocular 3D Human Pose Estimation. Adv. Neural Inf. Process. Syst..

[25] Chen H., Xu C., Zheng L., Zhang Q., Lin X. (2024). Diffusion-based graph generative methods. IEEE Trans. Knowl. Data Eng..

[26] Shan W., Zhang Y., Zhang X., Wang S., Zhou X., Ma S., Gao W. (2024). Diffusion-Based Hypotheses Generation and Joint-Level Hypotheses Aggregation for 3D Human Pose Estimation. IEEE Trans. Circuits Syst. Video Technol..

[27] Arthanari S., Jeong J.H., Joo Y.H. (2024). Exploiting multi-transformer encoder with multiple-hypothesis aggregation via diffusion model for 3D human pose estimation. Multimed. Tools Appl..

[28] Gu A., Dao T. (2023). Mamba: Linear-Time Sequence Modeling with Selective State Spaces. arXiv.

[29] Zhu L., Liao B., Zhang Q., Wang X., Liu W., Wang X. (2024). Vision Mamba: Efficient Visual Representation Learning with Bidirectional State Space Model. arXiv.

[30] Li K., Li X., Wang Y., He Y., Wang Y., Wang L., Qiao Y., Leonardis A., Ricci E., Roth S., Russakovsky O., Sattler T., Varol G. (2025). VideoMamba: State Space Model for Efficient Video Understanding. Computer Vision—ECCV 2024.

[31] Zhang B., Zhang P., Dong X., Zang Y., Wang J., Leonardis A., Ricci E., Roth S., Russakovsky O., Sattler T., Varol G. (2025). Long-CLIP: Unlocking the Long-Text Capability of CLIP. Computer Vision—ECCV 2024.

[32] Remelli E., Han S., Honari S., Fua P., Wang R. Lightweight multi-view 3D pose estimation through camera-disentangled representation. Proceedings of the IEEE/CVF Conference on Computer Vision and Pattern Recognition.

[33] Wu J., Ru X., Lu S., Xiong R., Wang Y. (2024). Recurrent Volume-based 3D Feature Fusion for Real-time Multi-view Object Pose Estimation. IEEE Trans. Instrum. Meas..

[34] Wang Y., Li M., Yan H. (2024). Utilizing motion segmentation for optimizing the temporal adjacency matrix in 3D human pose estimation. Neurocomputing.

[35] Wang Y., Wang Z., Li M., Yan H. 3d human pose estimation with two-step mixed-training strategy. Proceedings of the IEEE/CVF Winter Conference on Applications of Computer Vision.

[36] Zhou F., Yin J., Li P. (2024). Lifting by image–leveraging image cues for accurate 3d human pose estimation. Proc. AAAI Conf. Artif. Intell..

[37] Xu J., Guo Y., Peng Y. FinePOSE: Fine-grained prompt-driven 3d human pose estimation via diffusion models. Proceedings of the IEEE/CVF Conference on Computer Vision and Pattern Recognition.

[38] Wang Y., Li M., Meng N., Xu M. (2025). Optimizing the temporal adjacency matrix for 3D human pose estimation through clustering. Neurocomputing.

[39] Liu Y., Qiu C., Zhang Z. (2024). Deep learning for 3D human pose estimation and mesh recovery: A survey. Neurocomputing.

[40] Yu B.X., Zhang Z., Liu Y., Zhong S., Liu Y., Chen C.W. Gla-gcn: Global-local adaptive graph convolutional network for 3d human pose estimation from monocular video. Proceedings of the IEEE/CVF International Conference on Computer Vision.

[41] Li W., Liu H., Tang H., Wang P., Van Gool L. Mhformer: Multi-hypothesis transformer for 3d human pose estimation. Proceedings of the IEEE/CVF Conference on Computer Vision and Pattern Recognition.

[42] Li W., Liu M., Liu H., Wang P., Cai J., Sebe N. Hourglass Tokenizer for Efficient Transformer-Based 3D Human Pose Estimation. Proceedings of the 2024 IEEE/CVF Conference on Computer Vision and Pattern Recognition (CVPR).

[43] Einfalt M., Ludwig K., Lienhart R. Uplift and Upsample: Efficient 3D Human Pose Estimation with Uplifting Transformers. Proceedings of the 2023 IEEE/CVF Winter Conference on Applications of Computer Vision (WACV).

[44] Li W., Liu M., Liu H., Guo T., Wang T., Tang H., Sebe N. (2025). GraphMLP: A graph MLP-like architecture for 3D human pose estimation. Pattern Recognit..

[45] Mehraban S., Adeli V., Taati B. MotionAGFormer: Enhancing 3D Human Pose Estimation with a Transformer-GCNFormer Network. Proceedings of the IEEE/CVF Winter Conference on Applications of Computer Vision.

[46] Erol M.H., Senocak A., Feng J., Chung J.S. (2024). Audio mamba: Bidirectional state space model for audio representation learning. IEEE Signal Process. Lett..

[47] Ali A., Zimerman I., Wolf L. (2024). The hidden attention of mamba models. arXiv.

[48] Patro B.N., Agneeswaran V.S. (2024). Mamba-360: Survey of state space models as transformer alternative for long sequence modelling: Methods, applications, and challenges. arXiv.

[49] Xiao C., Cao Q., Luo Z., Lan L. MambaTrack: A Simple Baseline for Multiple Object Tracking with State Space Model. Proceedings of the 32nd ACM International Conference on Multimedia.

[50] Liu Y., Tian Y., Zhao Y., Yu H., Xie L., Wang Y., Ye Q., Jiao J., Liu Y. (2024). VMamba: Visual State Space Model. arXiv.

[51] Giuliari F., Scarpellini G., Fiorini S., James S., Morerio P., Wang Y., Del Bue A. (2024). Positional diffusion: Graph-based diffusion models for set ordering. Pattern Recognit. Lett..

[52] Huang Y., Liu J., Xian K., Qiu R.C. (2025). PoseMamba: Monocular 3D Human Pose Estimation with Bidirectional Global-Local Spatio-Temporal State Space Model. Proc. AAAI Conf. Artif. Intell..

[53] Che M., Xie C., Pan X., Yang Y., Dong J., Luo S., Chang X., Wang Y. (2025). 3D human pose estimation in the sailing simulator based on somatosensory interaction. Intell. Mar. Technol. Syst..

[54] Lu Y., Wang J., Gao J., Gong R., Cai C., Yap K.-H. A Structure-aware and Motion-adaptive Framework for 3D Human Pose Estimation with Mamba. Proceedings of the IEEE/CVF International Conference on Computer Vision.

[55] Cui H., Hua W., Huang R., Jia S., Hayama T. SasMamba: A Lightweight Structure-Aware Stride State Space Model for 3D Human Pose Estimation. Proceedings of the IEEE/CVF Winter Conference on Applications of Computer Vision (WACV).

[56] Yoshiyasu Y., Sun L., Sagawa R. MeshMamba: State Space Models for Articulated 3D Mesh Generation and Reconstruction. Proceedings of the IEEE/CVF International Conference on Computer Vision (ICCV).

[57] Lu Y., Liu J., Zhang Y., Liu Y., Tian X. Prompt distribution learning. Proceedings of the IEEE/CVF Conference on Computer Vision and Pattern Recognition.

[58] Zhou K., Yang J., Loy C.C., Liu Z. Conditional prompt learning for vision-language models. Proceedings of the IEEE/CVF Conference on Computer Vision and Pattern Recognition.

[59] Zheng H., Li H., Shi B., Dai W., Wang B., Sun Y., Guo M., Xiong H. Actionprompt: Action-guided 3d human pose estimation with text and pose prompting. Proceedings of the 2023 IEEE International Conference on Multimedia and Expo (ICME).

[60] Ionescu C., Papava D., Olaru V., Sminchisescu C. (2013). Human3.6M: Large Scale Datasets and Predictive Methods for 3D Human Sensing in Natural Environments. IEEE Trans. Pattern Anal. Mach. Intell. (TPAMI).

[61] Mehta D., Rhodin H., Casas D., Fua P., Sotnychenko O., Xu W., Theobalt C. Monocular 3d human pose estimation in the wild using improved cnn supervision. Proceedings of the 2017 International Conference on 3D Vision (3DV).

[62] Shan W., Liu Z., Zhang X., Wang Z., Han K., Wang S., Ma S., Gao W. Diffusion-based 3d human pose estimation with multi-hypothesis aggregation. Proceedings of the IEEE/CVF International Conference on Computer Vision.

[63] Chen Y., Wang Z., Peng Y., Zhang Z., Yu G., Sun J. Cascaded Pyramid Network for Multi-Person Pose Estimation. Proceedings of the IEEE/CVF Conference on Computer Vision and Pattern Recognition.

[64] Zhou J., Zhang T., Hayder Z., Petersson L., Harandi M. Diff3dhpe: A diffusion model for 3d human pose estimation. Proceedings of the IEEE/CVF International Conference on Computer Vision Workshops.

[65] Rommel C., Valle E., Chen M., Khalfaoui S., Marlet R., Cord M., Pérez P. Diffhpe: Robust, coherent 3d human pose lifting with diffusion. Proceedings of the IEEE/CVF International Conference on Computer Vision Workshops.

[66] Cai Q., Hu X., Hou S., Yao L., Huang Y. (2024). Disentangled diffusion-based 3d human pose estimation with hierarchical spatial and temporal denoiser. Proc. AAAI Conf. Artif. Intell..

[67] Chen T., Fang C., Shen X., Zhu Y., Chen Z., Luo J. (2022). Anatomy-aware 3d human pose estimation with bone-based pose decomposition. IEEE Trans. Circuits Syst. Video Technol..

[68] Shan W., Liu Z., Zhang X., Wang S., Ma S., Gao W., Avidan S., Brostow G., Cissé M., Farinella G.M., Hassner T. (2022). P-STMO: Pre-trained Spatial Temporal Many-to-One Model for 3D Human Pose Estimation. Computer Vision—ECCV 2022.

[69] Liu J., Liu M., Liu H., Li W. (2025). TCPFormer: Learning Temporal Correlation with Implicit Pose Proxy for 3D Human Pose Estimation. Proc. AAAI Conf. Artif. Intell..

[70] Tang Z., Qiu Z., Hao Y., Hong R., Yao T. 3D Human Pose Estimation with Spatio-Temporal Criss-Cross Attention. Proceedings of the IEEE/CVF Conference on Computer Vision and Pattern Recognition.

[71] Sun K., Xiao B., Liu D., Wang J. Deep High-Resolution Representation Learning for Human Pose Estimation. Proceedings of the IEEE/CVF Conference on Computer Vision and Pattern Recognition.

